# Direct-fed microbial supplementation influences the bacteria community composition of the gastrointestinal tract of pre- and post-weaned calves

**DOI:** 10.1038/s41598-018-32375-5

**Published:** 2018-09-20

**Authors:** Bridget E. Fomenky, Duy N. Do, Guylaine Talbot, Johanne Chiquette, Nathalie Bissonnette, Yvan P. Chouinard, Martin Lessard, Eveline M. Ibeagha-Awemu

**Affiliations:** 1 Agriculture and Agri-Food Canada, Sherbrooke Research and Development Centre, Sherbrooke, Québec, J1M 0C8 Canada; 2 Département des Sciences Animales, Université Laval, Québec, Québec, G1V 0A6 Canada; 3 Department of Animal Science, McGill University, Ste-Anne-de Bellevue, Quebec, H9X 3V9 Canada

## Abstract

This study investigated the effect of supplementing the diet of calves with two direct fed microbials (DFMs) (*Saccharomyces cerevisiae* boulardii CNCM I-1079 (SCB) and *Lactobacillus acidophilus* BT1386 (LA)), and an antibiotic growth promoter (ATB). Thirty-two dairy calves were fed a control diet (CTL) supplemented with SCB or LA or ATB for 96 days. On day 33 (pre-weaning, n = 16) and day 96 (post-weaning, n = 16), digesta from the rumen, ileum, and colon, and mucosa from the ileum and colon were collected. The bacterial diversity and composition of the gastrointestinal tract (GIT) of pre- and post-weaned calves were characterized by sequencing the V3-V4 region of the bacterial 16S rRNA gene. The DFMs had significant impact on bacteria community structure with most changes associated with treatment occurring in the pre-weaning period and mostly in the ileum but less impact on bacteria diversity. Both SCB and LA significantly reduced the potential pathogenic bacteria genera, *Streptococcus* and *Tyzzerella_4* (FDR ≤ 8.49E-06) and increased the beneficial bacteria, *Fibrobacter* (FDR ≤ 5.55E-04) compared to control. Other potential beneficial bacteria, including *Rumminococcaceae UCG 005, Roseburia* and *Olsenella*, were only increased (FDR ≤ *1.30E-02*) by SCB treatment compared to control. Furthermore, the pathogenic bacterium, *Peptoclostridium*, was reduced (FDR = 1.58E-02) by SCB only while LA reduced (FDR = 1.74E-05) *Ruminococcus_*2. Functional prediction analysis suggested that both DFMs impacted (p < 0.05) pathways such as cell cycle, bile secretion, proteasome, cAMP signaling pathway, thyroid hormone synthesis pathway and dopaminergic synapse pathway. Compared to the DFMs, ATB had similar impact on bacterial diversity in all GIT sites but greater impact on the bacterial composition of the ileum. Overall, this study provides an insight on the bacteria genera impacted by DFMs and the potential mechanisms by which DFMs affect the GIT microbiota and may therefore facilitate development of DFMs as alternatives to ATB use in dairy calf management.

## Introduction

The microbiota composition of the gastrointestinal tract (GIT) influences the health outcome of animals as well as their productivity^1,2^. The diversity and composition of the GIT microbiota can be influenced by many factors including age, diet, feeding method (management), and feed additives^3,4^. In particular, diet plays pivotal roles on the composition of the GIT microbiota^5–7^. Furthermore, diet and the weaning process affect the development of the GIT and microbial colonization in calves during the early period of growth^8,9^. Calf GIT is rapidly colonized by the maternal and environmental microorganisms during and after birth^4,10^. Consequently, exposure to beneficial microbes in the early period of growth will have relevant roles in health outcome^11^. It has been shown that diet and feeding management can be used to manipulate the rumen microbiota in ruminants with long lasting effects^12^. Likewise, microbial colonization and subsequent fermentation processes in the rumen during the early period of growth was influenced by feeding (natural or artificial) practice^13^.

Direct fed microbials (DFMs) have been shown to provide health benefits to the host mainly by modulating the GIT microbiota in cattle or other ruminants, and humans^2,14,15^. By modifying the composition of the GIT microbiota, DFMs may contribute to optimize beneficial functions of GIT microbial communities such as digestion, production of vitamin K, promotion and development of the immune system, and detoxification of harmful chemicals resulting in improvement of GIT health^16^. While the diversity, composition, and complexity of calves GIT microbiota has been mostly derived from the analyses of fecal^17–19^ and rumen microbiota^20,21^, few studies have characterized the diversity and community composition in the different sections of the GIT of 5 years old cows and 10 months old sheep^22,23^.

Manipulating the microbiota of the GIT through supplementation with DFMs is an attractive approach to improve and maintain animal health^24,25^. DFMs including *Saccharomyces cerevisiae* and *Lactobacillus acidophilus* are naturally occurring microorganisms in the GIT^26,27^. Introducing *Saccharomyces cerevisiae* boulardii CNCM I-1079 (SCB) and *Lactobacillus acidophilus* BT1386 (LA) soon after birth could provide beneficial impact in the establishment of the GIT microbiota. An increase in the potentially beneficial phylum, Actinobacteria, and genera, Bifidobacterium and Collinsella, in the cecum and colon of yeast supplemented piglets^28^ has been observed. Also, *Lactobacillus* spp. and *Bifidobacteruim* spp. were increased following treatment with several *Lactobacillus* species in a simulator of human intestinal microbial ecosystem^29^. Furthermore, SCB significantly improved the growth of total lactobacilli in the GIT especially around the weaning period and improved colon morphology^30^. Our hypothesis was that supplementation of calf’s diet with SCB and LA will increase the colonisation and establishment of beneficial bacteria in the different GIT sites.

Therefore, the present study investigated the effect of feeding SCB and LA on the colonisation and development of the GIT microbiota, their effects on the composition of bacterial populations in different GIT sites and their potential mechanisms of action during the early period of calf’s growth.

## Results

### Data acquisition

**Figure 1:**
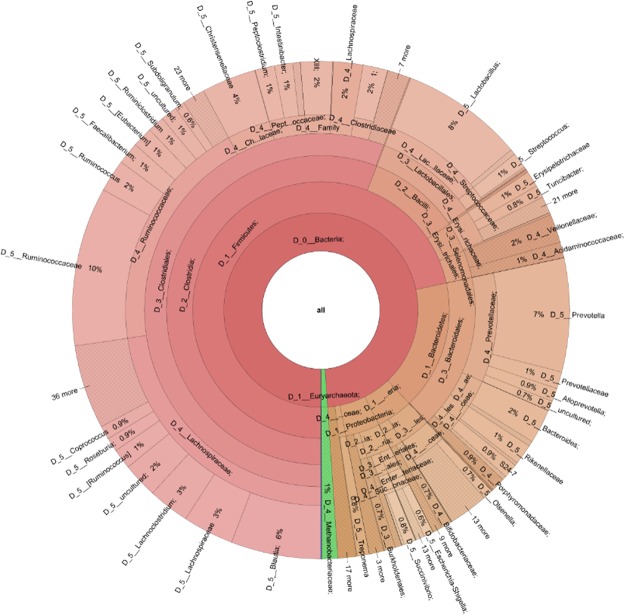
Distribution of 159 samples with complete 16S rRNA gene sequences of bacteria phylum and genera.

A total of 8,824,437 sequences of the 16S rRNA genes were generated from amplicon sequencing of 159 samples representing rumen (RuD), ileum (IlD) and colon (CoD) digesta and ileum (IlM) and colon (CoM) mucosa of 16 calves on day 33 (pre-weaning) and another 16 on day 96 (post-weaning) for a total of 4 calves per treatment (Control (CTL), SCB, LA, and an antibiotic growth promoter (ATB)). The mean number of sequences was 55,494.00 ± 1,969.00 per sample. A random sub-sample of sequences per sample were utilised for the normalisation of sequence numbers for other analyses. The sequencing depth was sufficient to cover each microbial community as shown on the rarefaction curves for each sample (Fig. S1). Overall, a total of 23 different phyla with 428 genera, 131 families, 81 order and 41 classes were detected (Fig. 1, Table S1a–e).

### Bacterial diversity across treatments in GIT sites at pre-weaning (day 33) and post-weaning (day 96)

A pairwise comparison of treatments was done within each GIT site on day 33 (pre-weaning) and day 96 (post-weaning) separately. The results of alpha diversity indices are shown in Table 1. In the pre-weaning period, ATB had bacterial communities with a tendency for a greater Shannon diversity index (p = 0.06) compared to CTL in IlM (Table 1). On the contrary, animals supplemented with ATB had bacterial communities with lower (p < 0.01) Shannon diversity index compared to that of CTL in RuD (Table 1). Moreover, SCB treatment had greater (p < 0.05) Simpson diversity index compared to ATB in CoM and greater bacterial richness (Chao1, p < 0.05) compared to ATB in CoD (Table 1). Meanwhile, LA had greater (p < 0.01) Shannon and Simpson diversity indices compared to ATB in CoM.

**Table 1:** Comparison of alpha diversity measures across treatments in gastrointestinal sites at pre- and post-weaning periods.

Gastrointestinal site	Alpha indices	Treatments^1^				P-value					
		CTRL	ATB	LA	SCB	ATB vs CTRL	ATB vs LA	ATB vs SCB	CTL vs LA	CTL vs SCB	LA vs SCB
**Pre-weaning (day 33)**											
Colon mucosa	Observed OTU	94.00	74.33	107.25	90.00	0.284	0.124	0.399	0.510	0.833	0.409
	Chao1	100.35	93.16	122.03	103.72	0.749	0.255	0.659	0.313	0.872	0.419
	Shannon	2.40	2.15	2.87	2.74	0.635	**0.004**	**0.065**	0.382	0.537	0.598
	Simpson	0.75	0.79	0.90	0.89	0.840	**0.005**	**0.021**	0.396	0.440	0.630
	InvSimpson	8.04	4.71	10.29	9.84	0.232	**0.006**	**0.047**	0.405	0.539	0.822
Colon digesta	Observed OTU	72.25	63.25	77.50	84.00	0.271	0.242	**0.092**	0.654	0.304	0.629
	Chao1	92.48	78.05	83.95	102.46	0.239	0.663	**0.014**	0.582	0.378	0.205
	Shannon	2.25	2.35	2.50	2.65	0.785	0.636	0.188	0.563	0.301	0.664
	Simpson	0.81	0.86	0.84	0.86	0.398	0.737	0.838	0.597	0.356	0.651
	InvSimpson	6.54	7.36	7.68	7.96	0.719	0.885	0.737	0.684	0.575	0.910
Ileum digesta	Observed OTU	61.00	71.75	70.25	53.25	0.484	0.920	0.212	0.462	0.445	0.132
	Chao1	76.86	91.30	89.90	70.55	0.382	0.938	0.242	0.419	0.660	0.264
	Shannon	1.25	1.05	1.44	1.42	0.739	0.512	0.516	0.681	0.688	0.978
	Simpson	0.50	0.43	0.55	0.58	0.755	0.621	0.516	0.821	0.663	0.819
	InvSimpson	2.34	2.74	2.72	3.07	0.741	0.992	0.814	0.663	0.500	0.771
Ileum mucosa	Observed OTU	67.50	105.50	84.50	103.75	**0.064**	0.280	0.940	0.326	0.146	0.416
	Chao1	82.90	113.53	91.46	107.25	**0.077**	0.172	0.764	0.604	0.294	0.480
	Shannon	1.19	2.51	1.84	1.71	**0.057**	0.398	0.246	0.413	0.449	0.867
	Simpson	0.45	0.78	0.60	0.55	**0.092**	0.411	0.264	0.532	0.637	0.853
	InvSimpson	2.34	8.13	5.63	2.99	0.216	0.614	0.261	0.339	0.578	0.432
Rumen digesta	Observed OTU	85.00	85.25	87.67	87.50	0.987	0.899	0.860	0.886	0.833	0.992
	Chao1	104.22	101.21	111.81	101.04	0.851	0.645	0.991	0.722	0.771	0.603
	Shannon	2.59	2.32	2.65	2.23	**0.004**	0.184	0.738	0.770	0.244	0.225
	Simpson	0.87	0.82	0.86	0.77	0.147	0.332	0.555	0.892	0.283	0.314
	InvSimpson	7.48	5.80	7.76	5.38	0.100	0.300	0.767	0.867	0.161	0.253
**Post –weaning (day 96)**											
Colon mucosa	Observed OTU	115.25	112.25	96.25	99.75	0.664	0.215	0.463	0.167	0.384	0.852
	Chao1	125.32	118.77	105.10	112.82	0.500	0.343	0.675	0.202	0.419	0.662
	Shannon	2.83	3.19	2.92	2.67	0.191	0.104	0.104	0.724	0.627	0.378
	Simpson	0.84	0.90	0.88	0.79	0.266	0.442	0.122	0.390	0.497	0.168
	InvSimpson	8.35	10.71	9.04	6.54	0.482	0.362	0.213	0.829	0.650	0.420
Colon digesta	Observed OTU	111.25	84.25	104.50	111.50	**0.019**	**0.046**	**0.017**	0.494	0.980	0.473
	Chao1	122.37	98.08	112.91	123.02	**0.069**	0.164	**0.064**	0.366	0.957	0.339
	Shannon	3.04	2.57	3.25	2.99	0.102	**0.041**	0.132	0.150	0.756	**0.089**
	Simpson	0.89	0.77	0.93	0.85	0.103	**0.053**	0.205	**0.005**	0.247	**0.049**
	InvSimpson	9.25	5.81	15.09	7.68	0.231	**0.022**	0.531	**0.001**	0.418	**0.013**
Ileum digesta	Observed OTU	80.75	73.75	60.00	79.00	0.737	0.287	0.693	0.330	0.932	0.145
	Chao1	93.67	87.95	73.56	102.88	0.818	0.386	0.423	0.424	0.719	0.128
	Shannon	2.53	2.27	2.17	2.54	0.529	0.686	0.155	0.412	0.970	0.175
	Simpson	0.84	0.81	0.78	0.88	0.673	0.594	**0.060**	0.462	0.580	0.165
	InvSimpson	10.12	5.56	5.59	8.30	0.301	0.988	**0.073**	0.316	0.657	0.210
Ileum mucosa	Observed OTU	103.00	95.25	75.25	94.25	0.507	0.209	0.945	0.120	0.589	0.314
	Chao1	110.33	102.59	87.11	103.01	0.569	0.194	0.974	0.149	0.652	0.291
	Shannon	2.60	2.52	1.99	1.84	0.878	0.478	0.377	0.352	0.271	0.847
	Simpson	0.82	0.77	0.64	0.60	0.782	0.517	0.490	0.315	0.350	0.869
	InvSimpson	7.87	9.18	6.26	4.47	0.759	0.584	0.288	0.711	0.224	0.674
Rumen digesta	Observed OTU	84.50	83.75	89.00	94.50	0.938	0.611	0.169	0.681	0.253	0.537
	Chao1	96.30	105.88	96.18	114.13	0.408	0.446	0.410	0.992	0.103	0.146
	Shannon	2.50	2.55	2.55	2.82	0.804	0.971	0.181	0.815	0.184	0.158
	Simpson	0.81	0.82	0.83	0.88	0.733	0.916	0.235	0.614	**0.051**	0.236
	InvSimpson	5.42	6.54	6.43	9.95	0.483	0.952	0.253	0.406	0.138	0.227

^1^Treatments: CTRL: Control fed milk replacer followed by starter feed, ATB: CTRL supplemented with antibiotics (ATB) chlortetracycline and neomycin (528 and 357 mg/L milk replacer, respectively), and chlortetracycline (55 mg/kg starter feed). LA: CTRL supplemented with *Lactobacillus acidophilus* BT1386 (LA; 2.5 × 10^8^ CFU/L milk replacer + 1 × 10^9^ CFU/kg starter feed) and SCB: CTRL supplemented with *Saccharomyces cerevisiae* boulardii CNCMI-1079 (SCB; 7.5 × 10^8^ colony forming units (CFU)/L milk replacer + 3 × 10^9^ CFU/kg starter feed).

In the post-weaning period, LA treatment had bacterial communities with greater (p < 0.01) Shannon, Simpson and InvSimpson diversity indices compared to ATB in CoD. SCB had bacterial communities with greater Simpson (p < 0.05) diversity index compared to CTL in RuD (Table 1).

**Figure 2:**
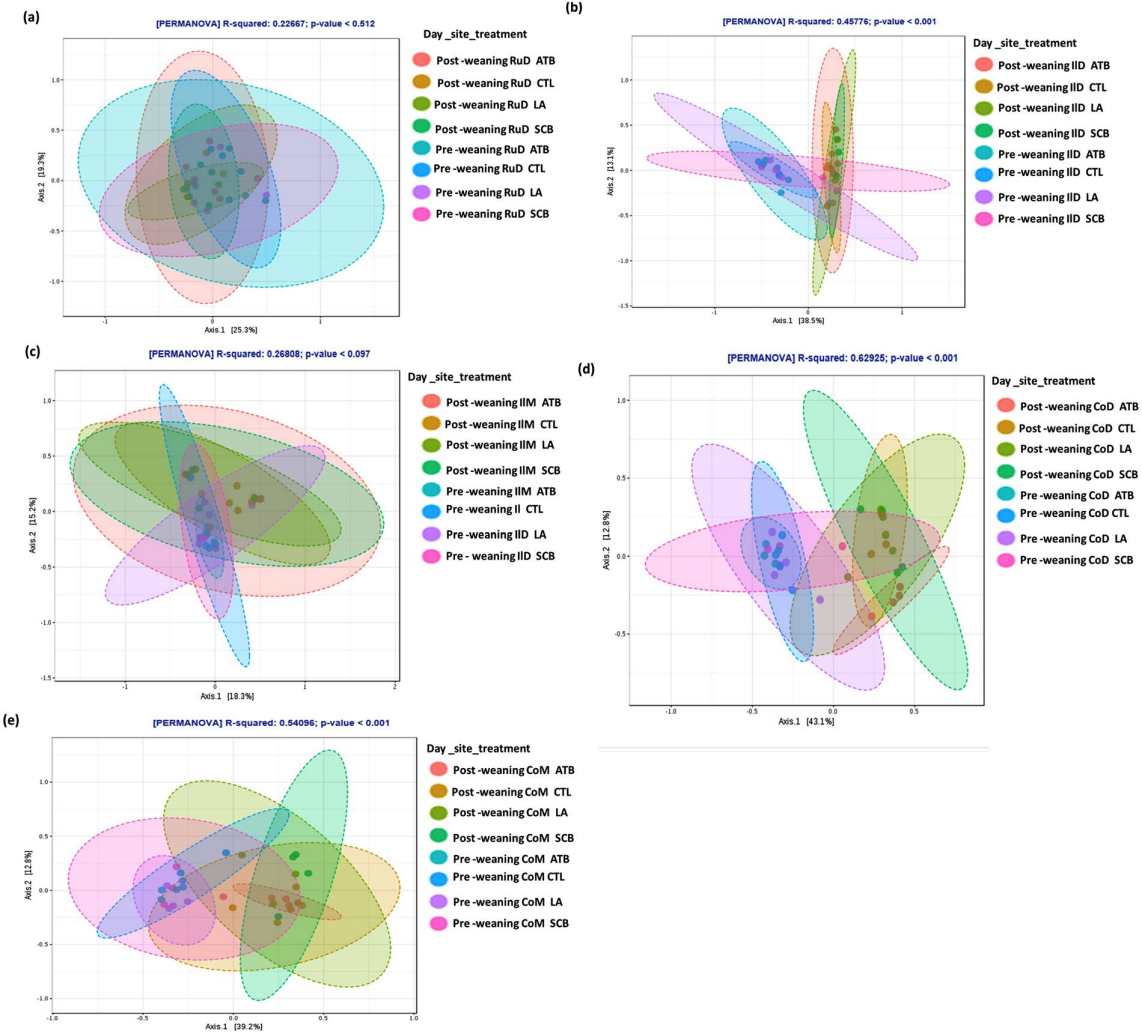
Principal coordinate analysis (PCoA) plots for treatment effect on each site at pre- and post-weaning periods. (**a**) Principal coordinate analysis (PCoA) plots for treatment effect on Rumen digesta at pre-weaning and post-weaning. (**b**) Principal coordinate analysis (PCoA) plots for treatment effect on ileum mucosa at pre-weaning and post-weaning. (**c**) Principal coordinate analysis (PCoA) plots for treatment effect on ileum digesta at pre-weaning and post-weaning. (**d**) Principal coordinate analysis (PCoA) plots for treatment effect on colon digesta at pre-weaning and post-weaning. (**e**) Principal coordinate analysis (PCoA) plots for treatment effect on colon mucosa at pre-weaning and post-weaning. Distances between the samples are based on similarity in OTU composition (OTU similarity 97%). A greater distance implies lower similarity, whereas similar OTUs will cluster together. The clustering pattern of the bacterial communities were tested using PERMANOVA and (*P* < 0.05) were considered significant.

For beta diversity, dissimilarities were mostly observed between periods, i.e. pre-weaning vs. post-weaning, as shown by the clustering pattern of the principal coordinate analysis (PCoA) plots at the different GIT sites (Fig. 2a–e). There was no dissimilarity (p = 0.512) in bacterial communities between treatments in RuD but a tendency (p = 0.09) was observed in IlM (Fig. 2a and c). However, there was a clear difference (p < 0.01) between all treatments in the pre-weaning period compared to the post-weaning period in IlD (Fig. 2b), CoD (Fig. 2d) and CoM (Fig. 2e).

### Bacterial composition and differential abundance across treatments in GIT sites at pre-weaning and post-weaning periods

**Figure 3:**
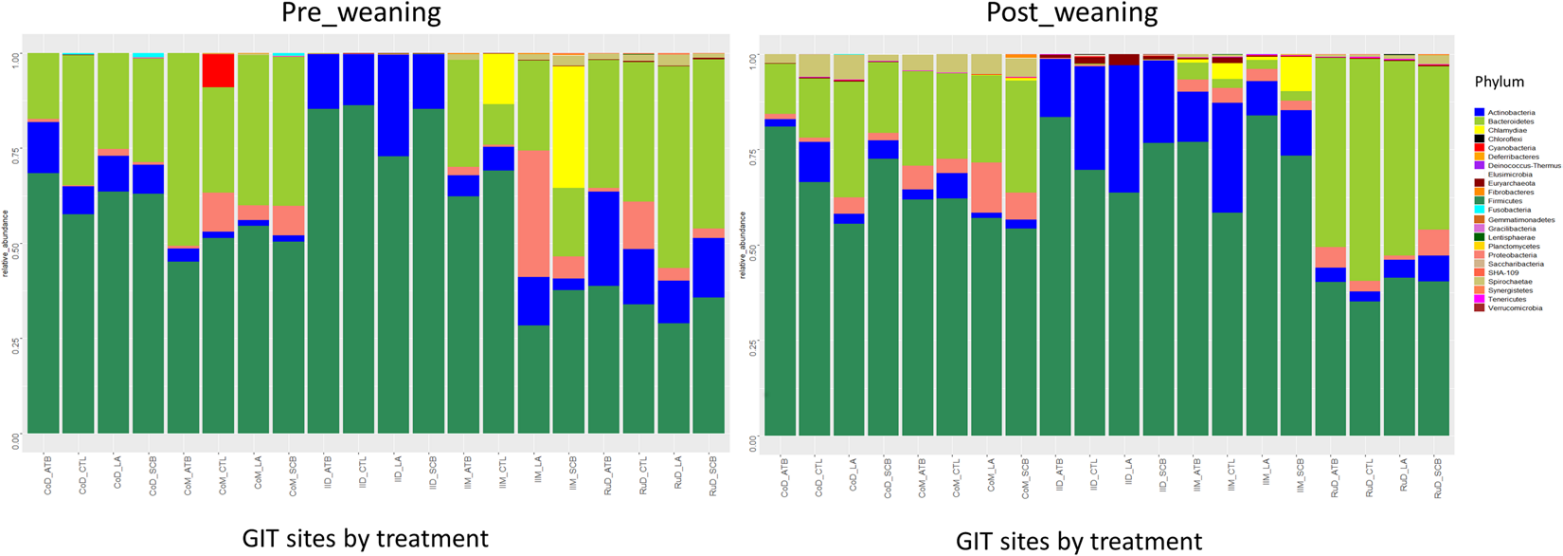
Stack bar charts of phylum level bacterial composition for the treatment effect on each site at pre- and post-weaning periods. CoM = colon mucosa, CoD = colon digesta, IM = ileum mucosa, IlD = ileum digesta, RuD = rumen digesta.

The most abundant phyla in all treatments (SCB, LA, ATB and CTL) at all GIT sites were either Firmicutes or Bacteriodetes at both pre- and post-weaning periods. However, Proteobacteria was the most abundant (33.31%) phylum in IlM for calves fed LA in the pre-weaning period (Fig. 3).

**Figure 4:**
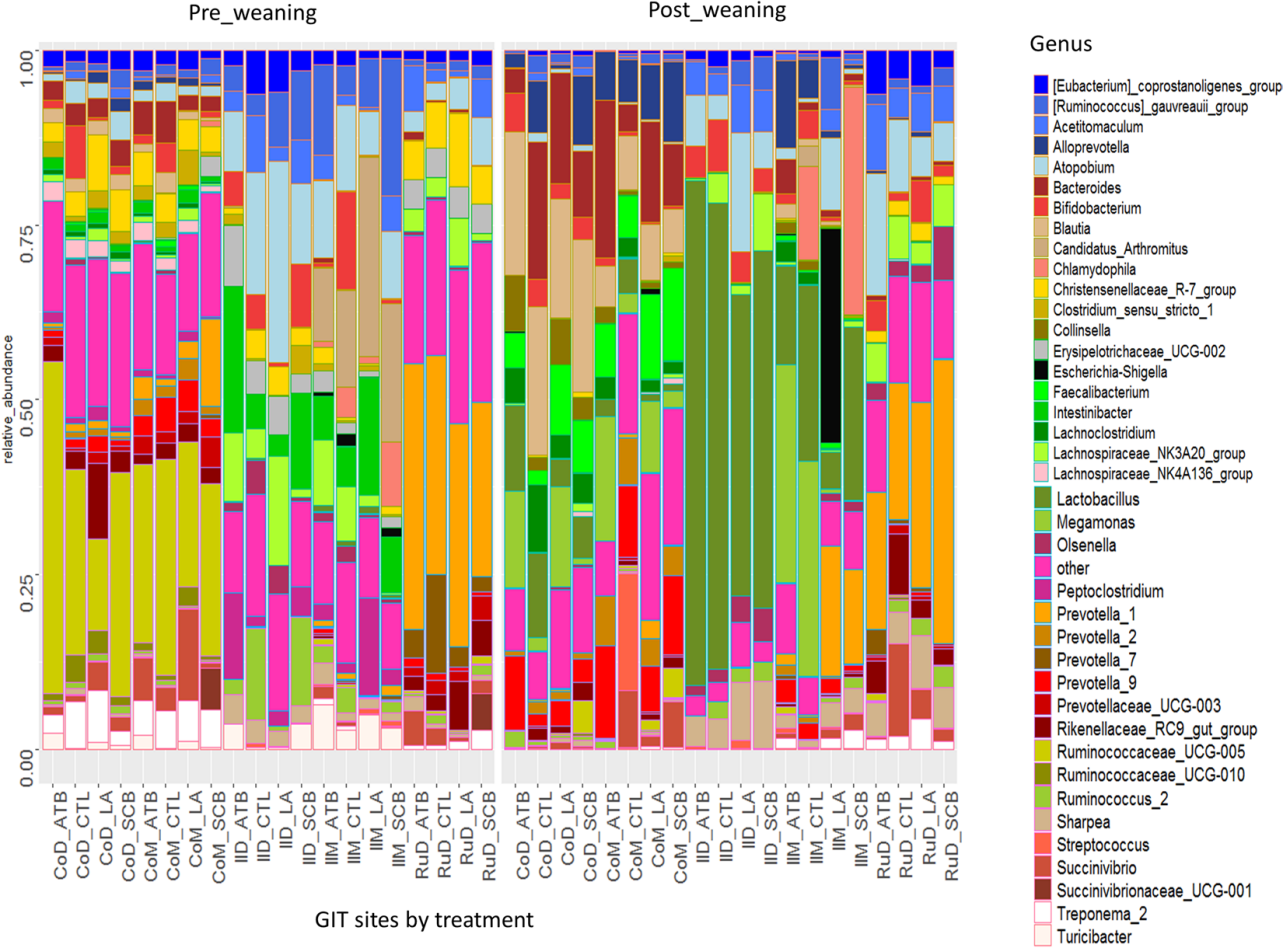
Stack bar charts of genus level bacterial composition for the treatment effect on each site at pre- and post-weaning periods. CoM = colon mucosa, CoD = colon digesta, IlM = ileum mucosa, IlD = ileum digesta, RuD = rumen digesta.

At the pre-weaning period, the most abundant genera for all treatments were *Blautia, Lactobacillus* and *Prevotella*_1 in CoD (17.1–21.9%), IlD (43.1–66.7%) and RuD (19.5–40.7%), respectively (Table S2). While the most abundant genera were *Bacteriodetes* for ATB (22.5%) and LA (14.3%), *Streptococcus* for CTL (16.7%) and *Faecalibacteria* for SCB (13.2%) in CoM (Fig. 4). The most abundant genera were *Megamonas* for CTL (30%) and ATB (31%), *Escherichia Shigella* for LA (30.7%) and *Chlamydophilia* for SCB (32.7%) in IlM (Fig. 4).

At the post-weaning period, *Ruminococcaceae*_UCG-005 was the most abundant genus in all treatments (13.2–47.5%) in CoD and CoM while *Atopobium* was the most abundant genus for both LA (28.8%) and CTL (17.5%) treatments and *Intestinibacter* for both ATB (20.9%) and SCB (13.6%) treatments in IlD. *Candidatus_Arthromitus* was the most dominant genus for both LA (28.60%) and SCB (19.9%) treatments while *Bifidobacterium* was the most abundant genus for CTL (14%) and *Ruminococcus_gauvreauii_group* for ATB (12.9%) in IlM (Fig. 4). *Prevotella_1* was the most abundant genus for all treatments (24.9–38.1%) in RuD.

**Figure 5:**
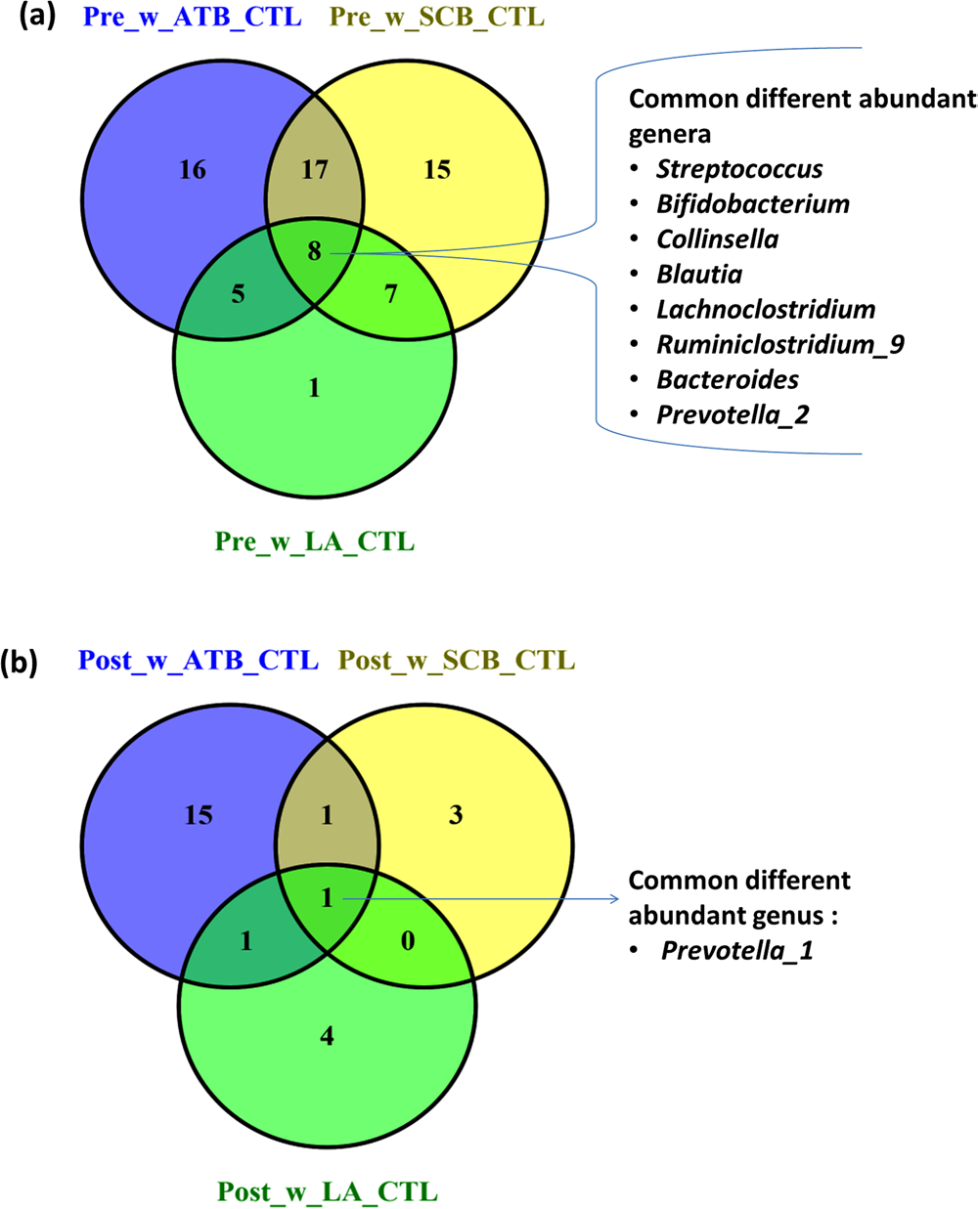
The common and specific genera in the (**a**) pre-weaning and (**b**) post-weaning periods for the different treatments.

Significant differential abundant (DA) genera between treatments (SCB, LA and ATB) and CTL in the pre- and post-weaning periods are shown in Tables 2, 3 and 4, respectively. The numbers of DA genera and common genera between the three pairwise comparisons are also shown in Fig. 5 for pre- and post-weaning periods. At the pre-weaning period, SCB significantly reduced the abundance of *Streptococcus* (FDR = 8.49E-06) and *Prevotella_7* (FDR = 1.49E-02) in CoM but increased (FDR = 1.30E-02) the abundance of *Ruminococcaceae_UCG-005* in CoD compared to CTL (Table 2). SCB treatment also significantly changed the relative abundance of 42 and two genera in IlM and IlD, respectively, but had no impact on the relative abundance of genera in RuD at the pre-weaning period. In IlM, the genera *Tyzzerella_4* (FDR = 4.27E-09) and *Ruminococcaceae_UCG-008* (FDR = 2.38E-04) had the highest log fold change reduction, while *Fibrobacter* (FDR = 5.5E-04) and *Roseburia* (FDR = 7.01E-04) had the highest log fold change increase by SCB compared to CTL. In IlD, *Ruminiclostridium_5* and *Christensenellaceae_R-7* genera were two genera significantly reduced (FDR = 2.52E-02) by SCB compared to CTL in the pre-weaning period.

**Table 2:** Significant differential abundant genera between control and SCB on day 33 (pre- weaning) and day 96 (post- weaning).

Gastrointestinal site	Genus	Phylum	Base Mean	L2FC^1^	P-value	FDR^2^
**Pre-weaning (day 33)**						
Colon mucosa	*Prevotella_7*	*Bacteroidetes*	215.29	7.98	1.50E-04	1.49E-02
	*Streptococcus*	*Firmicutes*	649.99	10.13	4.29E-08	8.49E-06
Colon digesta	*Ruminococcaceae_UCG-005*	*Firmicutes*	827.05	−7.44	6.55E-05	1.30E-02
Ileum Mucosa	*Acidaminococcus*	*Firmicutes*	10.78	−6.50	7.81E-03	3.00E-02
	*Bacteroides*	*Bacteroidetes*	5956.66	5.23	1.61E-03	7.92E-03
	*Bifidobacterium*	*Actinobacteria*	5947.20	7.02	5.47E-05	7.01E-04
	*Collinsella*	*Actinobacteria*	3794.58	7.39	3.42E-05	5.55E-04
	*Olsenella*	*Actinobacteria*	1082.09	−5.16	6.18E-04	4.46E-03
	*Desulfovibrio*	*Proteobacteria*	105.72	−7.76	1.02E-04	1.06E-03
	*Erysipelotrichaceae_UCG-001*	*Firmicutes*	54.14	−6.10	8.42E-04	5.12E-03
	*Erysipelatoclostridium*	*Firmicutes*	70.82	6.67	1.05E-03	5.70E-03
	*Erysipelotrichaceae_UCG-002*	*Firmicutes*	208.05	6.39	1.63E-03	7.92E-03
	*[Eubacterium]_nodatum_group*	*Firmicutes*	134.12	−4.11	1.33E-03	6.94E-03
	*Mogibacterium*	*Firmicutes*	22.25	−5.22	2.53E-03	1.12E-02
	*Fibrobacter*	*Fibrobacteres*	61.34	−10.57	3.38E-05	5.55E-04
	*Tyzzerella_4*	*Firmicutes*	1532.14	14.77	2.92E-11	4.27E-09
	*Lachnoclostridium*	*Firmicutes*	5966.51	8.95	9.48E-09	6.92E-07
	*Dorea*	*Firmicutes*	160.16	8.90	2.54E-05	5.55E-04
	*Roseburia*	*Firmicutes*	521.27	−6.67	5.03E-05	7.01E-04
	*Lachnospiraceae_NK3A20_group*	*Firmicutes*	1293.21	−5.75	6.42E-04	4.46E-03
	*Acetitomaculum*	*Firmicutes*	2437.10	−5.41	1.01E-03	5.65E-03
	*Howardella*	*Firmicutes*	24.17	−5.11	2.07E-03	9.73E-03
	*Blautia*	*Firmicutes*	4016.00	4.58	2.69E-03	1.16E-02
	*Lachnospiraceae_UCG-004*	*Firmicutes*	106.46	5.05	1.17E-02	4.08E-02
	*Peptoclostridium*	*Firmicutes*	119.32	5.21	4.02E-03	1.58E-02
	*Butyricimonas*	*Bacteroidetes*	10.20	−7.99	3.08E-03	1.25E-02
	*Prevotella_2*	*Bacteroidetes*	2892.26	7.99	8.61E-06	3.14E-04
	*Prevotella_1*	*Bacteroidetes*	6592.34	−6.15	6.24E-05	7.01E-04
	*Prevotellaceae_UCG-003*	*Bacteroidetes*	276.10	−6.41	7.67E-04	5.09E-03
	*Prevotellaceae_UCG-001*	*Bacteroidetes*	222.20	−6.44	9.23E-04	5.39E-03
	*Rikenellaceae_RC9_gut_group*	*Bacteroidetes*	1036.14	−5.21	4.28E-04	3.68E-03
	*Ruminococcaceae_UCG-008*	*Firmicutes*	1016.82	9.13	4.89E-06	2.38E-04
	*Pseudoflavonifractor*	*Firmicutes*	135.88	8.84	2.92E-05	5.55E-04
	*Ruminiclostridium_9*	*Firmicutes*	362.16	7.40	2.46E-05	5.55E-04
	*Ruminococcus_2*	*Firmicutes*	656.41	−5.04	4.63E-04	3.75E-03
	*Ruminococcus_1*	*Firmicutes*	223.20	−6.04	8.17E-04	5.12E-03
	*Ruminococcaceae_UCG-010*	*Firmicutes*	105.32	−6.67	2.78E-03	1.16E-02
	*[Eubacterium]_coprostanoligenes_group*	*Firmicutes*	1381.11	−3.68	8.32E-03	3.11E-02
	*Ruminococcaceae_UCG-014*	*Firmicutes*	171.47	−3.91	9.17E-03	3.35E-02
	*Ruminococcaceae_NK4A214_group*	*Firmicutes*	234.29	−4.49	9.94E-03	3.54E-02
	*Treponema_2*	*Spirochaetae*	1088.68	−7.03	6.08E-05	7.01E-04
	*Sphaerochaeta*	*Spirochaetae*	32.86	−9.86	1.34E-04	1.31E-03
	*Succinivibrio*	*Proteobacteria*	2214.12	−6.38	4.23E-04	3.68E-03
	*Veillonellaceae_UCG-001*	*Firmicutes*	62.99	−9.46	5.44E-04	4.18E-03
	*Megasphaera*	*Firmicutes*	264.01	−6.21	2.47E-03	1.12E-02
Ileum digesta	*Christensenellaceae_R-7_group*	*Firmicutes*	896.60	5.89	2.54E-04	2.52E-02
	*Ruminiclostridium_5*	*Firmicutes*	144.45	6.65	1.45E-04	2.52E-02
**Post weaning(day96)**						
Rumen digesta	*Ruminococcaceae_UCG-008*	*Firmicutes*	1016.82	9.13	6.68E-05	1.32E-02
Ileum mucosa	*Prevotella_1*	*Bacteroidetes*	6592.35	−5.95	0.000266	2.24E-02
	*Actinomyces*	*Actinobacteria*	10.95	−8.02	0.000293	2.24E-02
	*Streptococcus*	*Firmicutes*	649.99	−6.72	0.000407	2.24E-02
	*Rothia*	*Actinobacteria*	13.17	−8.23	0.000452	2.24E-02

^1^L2FC: log2fold change: positive (+) value indicates a decrease in relative abundance in SCB compared to control while negative value (−) indicates an increase in relative abundance in SCB compared to control,

^2^FDR: P value corrected for False Discovery Rate:

SCB: *Saccharomyces cerevisiae* boulardii CNCMI-1079 (SCB; 7.5 × 10^8^ colony forming units (CFU)/L milk replacer + 3 × 10^9^ CFU/kg starter feed).

**Table 3:** Significant differential abundant genera between control and LA on day 33 (pre -weaning) and day 96 (post- weaning).

Gastrointestinal site	Genera	Phylum	Base Mean	L2FC^1^	P-value	FDR^2^
**Pre-weaning (day 33)**						
Colon mucosa	*Turicibacter*	*Firmicutes*	67.38	6.41	4.84E-04	3.19E-02
	*Methylobacterium*	*Proteobacteria*	84.95	8.82	2.37E-04	2.35E-02
	*Streptococcus*	*Firmicutes*	649.99	9.39	2.25E-07	4.45E-05
Ileum mucosa	*Phascolarctobacterium*	*Firmicutes*	1075.81	7.18	1.92E-04	2.33E-03
	*Bacteroides*	*Bacteroidetes*	5956.66	5.78	1.07E-03	1.04E-02
	*Bifidobacterium*	*Actinobacteria*	5947.20	6.71	2.82E-04	3.16E-03
	*Collinsella*	*Actinobacteria*	3794.58	9.50	9.73E-07	2.84E-05
	*Erysipelatoclostridium*	*Firmicutes*	70.82	11.12	1.29E-06	3.13E-05
	*Fibrobacter*	*Fibrobacteres*	61.34	−7.72	3.60E-03	3.09E-02
	*Tyzzerella_4*	*Firmicutes*	1532.14	14.25	4.13E-10	6.02E-08
	*Lachnoclostridium*	*Firmicutes*	5966.51	9.25	3.44E-08	1.67E-06
	*Blautia*	*Firmicutes*	4016.00	7.37	8.27E-06	1.51E-04
	*Lachnospiraceae_UCG-004*	*Firmicutes*	106.46	8.90	9.22E-05	1.50E-03
	*Dorea*	*Firmicutes*	160.16	8.44	1.41E-04	2.06E-03
	*Intestinibacter*	*Firmicutes*	189.47	6.16	2.35E-03	2.14E-02
	*Prevotella_2*	*Bacteroidetes*	2892.26	8.88	4.57E-06	9.53E-05
	*Prevotella_9*	*Bacteroidetes*	6462.15	6.66	1.80E-04	2.33E-03
	*Ruminococcaceae_UCG-008*	*Firmicutes*	1016.82	13.16	7.42E-09	5.42E-07
	*Pseudoflavonifractor*	*Firmicutes*	135.88	11.74	3.30E-07	1.21E-05
	*Ruminiclostridium_9*	*Firmicutes*	362.16	6.02	1.02E-03	1.04E-02
	*Candidatus_Soleaferrea*	*Firmicutes*	22.07	7.88	3.89E-03	3.16E-02
**Post weaning (day 96)**						
Ileum digesta	*Ruminococcus_2*	*Firmicutes*	656.41	7.45	3.95E-07	1.74E-05
	*Lactobacillus*	*Firmicutes*	19739.88	5.74	5.19E-04	8.93E-03
	*Ruminiclostridium_9*	*Firmicutes*	362.16	5.98	7.77E-04	8.93E-03
	*Prevotella_1*	*Bacteroidetes*	6592.35	5.75	8.12E-04	8.93E-03
	*Acetitomaculum*	*Firmicutes*	2437.10	5.15	2.13E-03	1.88E-02
	*Ruminococcaceae_NK4A214_group*	*Firmicutes*	234.29	4.48	3.36E-03	2.46E-02

^1^L2FC: log2fold change, positive (+) value indicates a decrease in relative abundance in LA compared to control while negative value (−) indicates increase in relative abundance in LA compared to control.

^2^FDR: p values corrected for False Discovery Rate.

LA: CTRL supplemented with *Lactobacillus acidophilus* BT1386 (LA; 2.5 × 10^8^ CFU/L milk replacer.

**Table 4:** Significant differential abundant genera between control and ATB on day 33 (pre -weaning) and day 96 (post-weaning) periods.

Gastrointestinal site	Genus	Phylum	Base Mean	L2FC^1^	P-value	FDR^2^
**Pre-weaning(day 33)**						
Colon mucosa	*Streptococcus*	*Firmicutes*	649.99	8.06	3.01E-05	5.97E-03
Ileum digesta	*Actinomyces*	*Actinobacteria*	10.95	−5.69	4.04E-03	2.16E-02
	*Bifidobacterium*	*Actinobacteria*	5947.20	−5.76	8.48E-04	6.93E-03
	*Olsenella*	*Actinobacteria*	1082.09	−5.62	2.64E-05	4.59E-04
	*Atopobium*	*Actinobacteria*	1724.26	−3.36	1.24E-03	9.08E-03
	*Collinsella*	*Actinobacteria*	3794.58	−4.94	7.68E-03	3.56E-02
	*Desulfovibrio*	*Proteobacteria*	105.72	−12.62	4.34E-10	6.03E-08
	*Erysipelotrichaceae_UCG-001*	*Firmicutes*	54.14	−6.90	4.78E-06	1.59E-04
	*Turicibacter*	*Firmicutes*	67.38	−7.91	1.27E-04	1.96E-03
	*Sharpea*	*Firmicutes*	2807.21	−4.69	4.08E-04	4.05E-03
	*[Eubacterium]_nodatum_group*	*Firmicutes*	134.12	−3.80	1.53E-04	2.13E-03
	*Mogibacterium*	*Firmicutes*	22.25	−3.97	3.10E-03	1.79E-02
	*Roseburia*	*Firmicutes*	521.27	−7.21	2.20E-07	1.53E-05
	*Syntrophococcus*	*Firmicutes*	285.49	−6.50	5.42E-07	2.51E-05
	*Blautia*	*Firmicutes*	4016.00	−7.25	8.44E-06	1.96E-04
	*Acetitomaculum*	*Firmicutes*	2437.10	−5.21	9.25E-04	7.14E-03
	*Howardella*	*Firmicutes*	24.17	−3.53	7.96E-03	3.57E-02
	*Lachnoclostridium*	*Firmicutes*	5966.51	−3.99	9.61E-03	4.05E-02
	*Methanosphaera*	*Euryarchaeota*	24.49	−5.18	2.18E-04	2.76E-03
	*Methylobacterium*	*Proteobacteria*	84.95	−7.00	2.43E-03	1.52E-02
	*Peptoclostridium*	*Firmicutes*	119.32	−5.38	2.51E-03	1.52E-02
	*Romboutsia*	*Firmicutes*	10.11	−7.48	2.38E-03	1.52E-02
	*Intestinibacter*	*Firmicutes*	189.47	−5.03	4.23E-03	2.18E-02
	*Prevotella_1*	*Bacteroidetes*	6592.35	−5.11	2.13E-03	1.48E-02
	*Rikenellaceae_RC9_gut_group*	*Bacteroidetes*	1036.14	−4.54	7.55E-03	3.56E-02
	*Ruminococcus_1*	*Firmicutes*	223.20	−7.60	5.71E-06	1.59E-04
	*Ruminococcaceae_NK4A214_group*	*Firmicutes*	234.29	−5.26	2.75E-04	3.18E-03
	*Ruminiclostridium*	*Firmicutes*	6.81	−8.06	3.43E-04	3.67E-03
	*[Eubacterium]_coprostanoligenes_group*	*Firmicutes*	1381.11	−4.61	5.57E-04	5.16E-03
	*Ruminococcaceae_UCG-002*	*Firmicutes*	149.04	−6.54	6.98E-04	6.06E-03
	*Anaerotruncus*	*Firmicutes*	115.29	−5.59	6.06E-03	3.01E-02
	*Ruminiclostridium_9*	*Firmicutes*	362.16	4.97	8.34E-03	3.62E-02
	*Treponema_2*	*Spirochaetae*	1088.68	−5.80	3.69E-03	2.05E-02
	*Cloacibacillus*	*Synergistetes*	16.61	−7.12	1.14E-02	4.67E-02
	*Megasphaera*	*Firmicutes*	264.01	−8.18	2.45E-05	4.59E-04
Rumen digesta	*Phascolarctobacterium*	*Firmicutes*	1075.81	−5.82	3.84E-03	2.91E-02
	*Bacteroides*	*Bacteroidetes*	5956.66	−6.36	6.64E-04	1.88E-02
	*Bifidobacterium*	*Actinobacteria*	5947.20	−6.37	1.01E-03	1.88E-02
	*Olsenella*	*Actinobacteria*	1082.09	−4.82	1.49E-03	1.88E-02
	*Atopobium*	*Actinobacteria*	1724.26	−3.57	2.70E-03	2.75E-02
	*Elusimicrobium*	*Elusimicrobia*	4.43	−7.96	3.87E-03	2.91E-02
	*Erysipelotrichaceae_UCG-001*	*Firmicutes*	54.14	−4.51	6.93E-03	4.38E-02
	*Mogibacterium*	*Firmicutes*	22.25	−6.05	1.05E-04	7.67E-03
	*[Eubacterium]_brachy_group*	*Firmicutes*	19.74	−7.00	1.51E-03	1.88E-02
	*[Eubacterium]_hallii_group*	*Firmicutes*	28.17	−7.45	8.55E-04	1.88E-02
	*Blautia*	*Firmicutes*	4016.00	−5.89	1.55E-03	1.88E-02
	*Syntrophococcus*	*Firmicutes*	285.49	−4.71	1.35E-03	1.88E-02
	*Lachnospiraceae_UCG-008*	*Firmicutes*	12.39	−6.20	2.83E-03	2.75E-02
	*Acetitomaculum*	*Firmicutes*	2437.10	−5.11	3.99E-03	2.91E-02
	*Lachnospiraceae_NK3A20_group*	*Firmicutes*	1293.21	−5.43	3.20E-03	2.91E-02
	*Methanosphaera*	*Euryarchaeota*	24.49	−7.09	8.00E-06	1.17E-03
	*Prevotella_2*	*Bacteroidetes*	2892.26	−7.17	1.53E-03	1.88E-02
	*Prevotella_9*	*Bacteroidetes*	6462.15	−5.24	5.21E-03	3.45E-02
	*Ruminococcaceae_UCG*−013	*Firmicutes*	27.66	−8.91	2.77E-04	1.35E-02
	*[Eubacterium]_coprostanoligenes_group*	*Firmicutes*	1381.11	−5.19	6.23E-04	1.88E-02
	*Ruminiclostridium*	*Firmicutes*	6.81	−7.71	1.85E-03	2.08E-02
	*Ruminococcaceae_UCG-002*	*Firmicutes*	149.04	−5.60	4.56E-03	3.17E-02
	*Streptococcus*	*Firmicutes*	649.99	−5.44	7.20E-03	4.38E-02
	*Ruminobacter*	*Proteobacteria*	450.64	−7.89	3.45E-03	2.91E-02
**Post- weaning(day 96)**						
Ileum digesta	*Ruminococcus_2*	*Firmicutes*	656.41	5.11	1.99E-04	2.56E-02
	*Ruminococcaceae_UCG-008*	*Firmicutes*	1016.82	7.88	2.59E-04	2.56E-02
Ileum mucosa	*Dorea*	*Firmicutes*	160.16	−9.10	3.40E-05	2.74E-03
	*Sutterella*	*Proteobacteria*	99.95	−7.83	5.92E-05	2.74E-03
	*Prevotellaceae_UCG-003*	*Bacteroidetes*	276.10	−7.40	8.56E-05	2.74E-03
	*Rikenellaceae_RC9_gut_group*	*Bacteroidetes*	1036.14	−5.99	1.02E-04	2.74E-03
	*Anaerovibrio*	*Firmicutes*	241.51	−7.61	2.41E-04	5.15E-03
	*Prevotella_1*	*Bacteroidetes*	6592.35	−5.62	5.81E-04	1.04E-02
	*Lachnoclostridium*	*Firmicutes*	5966.51	−5.55	8.85E-04	1.35E-02
	*Prevotella_9*	*Bacteroidetes*	6462.15	−5.70	1.34E-03	1.79E-02
	*Prevotella_2*	*Bacteroidetes*	2892.26	−6.00	1.50E-03	1.79E-02
	*Ruminococcaceae_UCG-005*	*Firmicutes*	827.05	−5.30	2.70E-03	2.89E-02
	*Treponema_2*	*Spirochaetae*	1088.68	−5.32	3.52E-03	3.42E-02
	*Ruminococcaceae_UCG-010*	*Firmicutes*	105.32	−5.95	4.65E-03	4.15E-02
	*Ruminococcaceae_UCG-009*	*Firmicutes*	15.20	−7.25	5.71E-03	4.68E-02
	*Succinivibrio*	*Proteobacteria*	2214.12	−5.18	6.19E-03	4.68E-02
	*Lachnospiraceae_NK4A136_group*	*Firmicutes*	95.27	−5.57	6.56E-03	4.68E-02
	*Prevotella_7*	*Bacteroidetes*	215.29	5.90	7.29E-03	4.88E-02

^1^L2FC: log2fold change log 2 fold change, positive (+) value indicates a decrease in relative abundance in control compared to ATB while negative value (−) indicates increase in relative abundance in ATB compared to control.

^2^FDR: P value corrected for False Discovery Rate.

ATB: chlortetracycline and neomycin (528 and 357 mg/L milk replacer, respectively), and chlortetracycline (55 mg/kg starter feed).

In the post weaning period, SCB significantly reduced the abundance of *Ruminococcaceae_UCG-008* in RuD (FDR = 1.32E-02) but increased (FDR = 2.24E-02) the relative abundance of four genera (*Prevotella_1, Actinomycetes, Streptococcus* and *Rothia*) in IlM compared to CTL. Genera relative abundance in other sites was not affected by SCB in the post-weaning period (Table 2).

In the pre-weaning period, no genus was significantly affected by LA treatment in the RuD, llD and CoD compared to CTL, but three and 18 genera were significantly affected in CoM and IlM respectively. In llM, *Tyzzerella_4, Ruminococcaceae_UCG-008* and *Lachnoclostridium* were the top three genera significantly reduced (FDR ≤ 1.67E-06) while *Fibrobacter* was significantly increased (FDR = 3.09E-02) by LA treatment compared to CTL (Table 3). In the post-weaning period, LA treatment impacted only the IlD, by reducing (FDR ≤ 2.46E-02) the relative abundance of six genera (*Ruminococcus_2, Lactobacillus, Ruminiclostridium_9, Prevotella_1*, *Acetitomaculum and Ruminococcaceae_NKA214_group* (Table 3).

The ATB treatment had greater impact on genera relative abundance in llD and RuD at the pre-weaning period and in IlM at the post-weaning period (Table 4). ATB changed (FDR ≤ 9.08E-03) the relative abundance of 34 and 24 genera in IlD and RuD in the pre-weaning period and 16 genera in IlM. *Streptococcus* was significantly reduced (FDR = 5.97E-03) by ATB treatment in CoM at the pre-weaning period. In the post-weaning period, *Dorea* (FDR = 2.74E-03) and *Anaerovibrio* (FDR = 5.15E-03) were significantly increased by ATB (Table 4).

Comparisons between LA vs. ATB, SCB vs. ATB and SCB vs. LA are shown in Tables 5, 6 and S3. A total of 43 and 135 genera were significantly DA between LA vs. ATB (Table 5) and SCB vs. ATB (Table 6), respectively. Most DA genera for both pairwise comparisons were found in the pre-weaning period (40/43 for LA vs. ATB and 113/135 for SCB vs. ATB) as well as in the ileum (mucosa and digesta) (Tables 5 and 6). *Tyzzerella* 4 (FDR = 4.42E-11) and Ruminococcaceae_UCG-005 (FDR = 8.45E-07) were the most significant DA genera between SCB vs. ATB in the pre- and post-weaning period, respectively (Table 5). *Tyzzerella* 4 was also the most significant DA genus in the pre-weaning period when comparing LA vs. ATB (FDR = 7.91E-10) (Table 6).

**Table 5:** Highly significant differential abundant genera between SCB and ATB on day 33 (pre -weaning) and day 96 (post-weaning)^1^.

Gastrointestinal site	Genus	Phylum	Base Mean	L2FC^2^	P-value	FDR^3^
**Pre-weaning (day 33)**						
Ileum mucosa	Tyzzerella 4	Firmicutes	1532.14	16.85	3.32E-13	4.42E-11
	Lachnoclostridium	Firmicutes	5966.51	11.22	1.36E-11	9.04E-10
	Ruminococcaceae UCG-008	Firmicutes	1016.82	11.38	6.23E-08	2.56E-06
	Pseudoflavonifractor	Firmicutes	135.88	11.82	8.16E-08	2.56E-06
	Prevotella 2	Bacteroidetes	2892.26	10.14	9.63E-08	2.56E-06
	Ruminiclostridium 9	Firmicutes	362.16	9.75	1.38E-07	3.06E-06
	Collinsella	Actinobacteria	3794.58	9.19	1.20E-06	2.15E-05
	Bifidobacterium	Actinobacteria	5947.20	8.95	1.29E-06	2.15E-05
	Erysipelatoclostridium	Firmicutes	70.82	9.36	9.76E-06	1.44E-04
	Bacteroides	Bacteroidetes	5956.66	7.69	1.30E-05	1.73E-04
	Blautia	Firmicutes	4016.00	6.88	2.38E-05	2.87E-04
	Anaerotruncus	Firmicutes	115.29	6.98	7.22E-05	8.00E-04
	Subdoligranulum	Firmicutes	121.14	7.04	2.88E-04	2.83E-03
	Ruminococcus 1	Firmicutes	223.20	−6.80	3.18E-04	2.83E-03
	Lachnospiraceae FCS020 group	Firmicutes	7.11	7.67	3.19E-04	2.83E-03
	Lachnospiraceae UCG-004	Firmicutes	106.46	7.31	4.69E-04	3.90E-03
	Prevotella 1	Bacteroidetes	6592.34	−5.58	6.60E-04	5.16E-03
	Phascolarctobacterium	Firmicutes	1075.81	6.21	9.25E-04	6.84E-03
Ileum digesta	Desulfovibrio	Proteobacteria	105.72	10.35	1.47E-08	2.00E-06
	Ruminococcus 1	Firmicutes	223.20	8.65	2.70E-07	1.83E-05
	Lachnoclostridium	Firmicutes	5966.51	7.30	3.30E-06	1.50E-04
	Syntrophococcus	Firmicutes	285.49	5.74	8.76E-06	2.98E-04
	Methanosphaera	Euryarchaeota	24.49	5.85	3.00E-05	7.46E-04
	[Eubacterium] coprostanoligenes group	Firmicutes	1381.11	5.55	3.29E-05	7.46E-04
	Roseburia	Firmicutes	521.27	5.59	5.67E-05	1.06E-03
	Ruminococcaceae UCG-002	Firmicutes	149.04	8.32	6.23E-05	1.06E-03
	Intestinibacter	Firmicutes	189.47	7.55	7.65E-05	1.16E-03
	Streptococcus	Firmicutes	649.99	6.91	1.15E-04	1.57E-03
	Erysipelotrichaceae UCG-001	Firmicutes	54.14	5.61	1.45E-04	1.79E-03
	Atopobium	Actinobacteria	1724.26	3.83	2.38E-04	2.66E-03
	Methylobacterium	Proteobacteria	84.95	8.84	2.55E-04	2.66E-03
	Bifidobacterium	Actinobacteria	5947.20	6.24	3.01E-04	2.79E-03
	[Eubacterium] nodatum group	Firmicutes	134.12	3.62	3.08E-04	2.79E-03
	Ruminococcaceae NK4A214 group	Firmicutes	234.29	5.15	3.29E-04	2.80E-03
	Ruminococcaceae UCG-005	Firmicutes	827.05	6.72	5.24E-04	4.19E-03
	Turicibacter	Firmicutes	67.38	6.35	9.15E-04	6.91E-03
	Peptoclostridium	Firmicutes	119.32	5.81	1.08E-03	7.04E-03
	Lactobacillus	Firmicutes	19739.88	5.04	1.12E-03	7.04E-03
	Lachnospira	Firmicutes	13.66	8.73	1.15E-03	7.04E-03
	Christensenellaceae R-7 group	Firmicutes	896.60	5.28	1.15E-03	7.04E-03
	Sharpea	Firmicutes	2807.21	4.30	1.19E-03	7.04E-03
Rumen digesta	Prevotella 7	Bacteroidetes	215.29	9.80	3.16E-06	4.39E-04
	Ruminococcaceae UCG-005	Firmicutes	827.05	7.98	7.51E-06	5.22E-04
	Atopobium	Actinobacteria	1724.26	4.82	1.67E-05	7.72E-04
	Methanosphaera	Euryarchaeota	24.49	5.78	6.28E-05	2.14E-03
	Lachnospiraceae UCG-008	Firmicutes	12.39	7.98	8.59E-05	2.14E-03
	Streptococcus	Firmicutes	649.99	7.60	9.22E-05	2.14E-03
	[Eubacterium] coprostanoligenes group	Firmicutes	1381.11	5.51	1.15E-04	2.24E-03
	Ruminococcaceae NK4A214 group	Firmicutes	234.29	5.81	1.29E-04	2.24E-03
	Bifidobacterium	Actinobacteria	5947.20	6.72	2.49E-04	3.84E-03
	Lactobacillus	Firmicutes	19739.88	5.86	5.40E-04	7.33E-03
	Mogibacterium	Firmicutes	22.25	4.87	5.80E-04	7.33E-03
	Corynebacterium 1	Actinobacteria	33.17	8.08	6.50E-04	7.53E-03
Colon mucosa	Succinivibrio	Proteobacteria	2214.12	−8.17	1.84E-05	3.65E-03
	Tyzzerella 4	Firmicutes	1532.14	8.10	1.01E-04	9.98E-03
Colon digesta	Tyzzerella 4	Firmicutes	1532.14	8.08	2.59E-05	5.12E-03
**Post-weaning (day 96)**						
Ileum mucosa	Ruminococcaceae_UCG-005	Firmicutes	827.05	10.32	6.21E-09	8.45E-07
	Prevotellaceae_UCG-003	Bacteroidetes	276.10	9.30	8.50E-07	5.78E-05
	Dorea	Firmicutes	160.16	9.47	2.21E-06	8.60E-05
	Prevotella_7	Bacteroidetes	215.29	−9.86	2.53E-06	8.60E-05
	Anaerovibrio	Firmicutes	241.51	8.62	1.66E-05	3.96E-04
	Ruminococcaceae_UCG-010	Firmicutes	105.32	9.21	1.75E-05	3.96E-04
	Actinomyces	Actinobacteria	10.95	−8.84	3.36E-05	6.53E-04
	Phascolarctobacterium	Firmicutes	1075.81	7.90	5.58E-05	9.48E-04
	Lachnoclostridium	Firmicutes	5966.51	6.48	9.10E-05	1.37E-03
	Lachnospiraceae_NK4A136_group	Firmicutes	95.27	7.96	1.28E-04	1.74E-03
	Ruminiclostridium_9	Firmicutes	362.16	6.92	2.24E-04	2.68E-03
	Rothia	Actinobacteria	13.17	−8.46	2.36E-04	2.68E-03
	Prevotella_2	Bacteroidetes	2892.26	6.41	4.50E-04	4.71E-03
	Lachnospiraceae_UCG-005	Firmicutes	91.57	−8.64	8.16E-04	7.92E-03
	Faecalibacterium	Firmicutes	1438.15	5.82	1.06E-03	9.61E-03

^1^Results presented only for genera with FDR < 0.01; the complete results are presented in Table S3b.

ATB: chlortetracycline and neomycin (528 and 357 mg/L milk replacer, respectively), and chlortetracycline (55 mg/kg starter feed), SCB: *Saccharomyces cerevisiae* boulardii CNCMI-1079 (SCB; 7.5 × 10^8^ colony forming units (CFU)/L milk replacer + 3 × 10^9^ CFU/kg starter feed).

^2^L2FC: log2fold change, positive (+) value indicates a decrease in relative abundance in SCB compared to ATB, negative value (−) indicates increase in relative abundance in SCB compared to ATB.

^3^FDR: P value corrected for False Discovery Rate.

**Table 6:** Highly significant differential abundant genera between LA and ATB on day 33 (pre- weaning) and day 96 (post- weaning)^1^.

Gastrointestinal sites	Genus	Phylum	Base Mean	L2FC^2^	P-value	FDR^3^
**Pre-weaning (day 33)**						
Ileum mucosa	Tyzzerella 4	Firmicutes	1532.14	16.34	5.69E-12	7.91E-10
	Lachnoclostridium	Firmicutes	5966.51	11.52	7.50E-11	3.51E-09
	Ruminococcaceae UCG-008	Firmicutes	1016.82	15.41	7.57E-11	3.51E-09
	Pseudoflavonifractor	Firmicutes	135.88	14.71	6.26E-10	2.17E-08
	Erysipelatoclostridium	Firmicutes	70.82	13.80	5.40E-09	1.50E-07
	Collinsella	Actinobacteria	3794.58	11.29	3.04E-08	6.16E-07
	Blautia	Firmicutes	4016.00	9.67	3.10E-08	6.16E-07
	Prevotella 2	Bacteroidetes	2892.26	11.03	5.94E-08	1.03E-06
	Lachnospiraceae FCS020 group	Firmicutes	7.11	7.93	1.81E-04	1.68E-03
	Candidatus Soleaferrea	Firmicutes	22.07	10.23	2.47E-04	2.15E-03
	Subdoligranulum	Firmicutes	121.14	7.20	5.03E-04	4.11E-03
	Ruminococcaceae UCG-005	Firmicutes	827.05	6.99	6.38E-04	4.93E-03
Ileum digesta	Desulfovibrio	Proteobacteria	105.72	11.33	4.72E-09	6.42E-07
	Lachnospiraceae UCG-004	Firmicutes	106.46	11.16	2.08E-06	3.22E-05
	Phascolarctobacterium	Firmicutes	1075.81	9.47	2.92E-06	4.06E-05
	Prevotella 9	Bacteroidetes	6462.15	8.57	5.05E-06	6.38E-05
	Bifidobacterium	Actinobacteria	5947.20	8.64	9.44E-06	1.08E-04
	Bacteroides	Bacteroidetes	5956.66	8.24	1.01E-05	1.08E-04
	Ruminiclostridium 9	Firmicutes	362.16	8.37	1.38E-05	1.37E-04
	Roseburia	Firmicutes	521.27	6.40	4.26E-06	2.65E-04
	Ruminococcus 1	Firmicutes	223.20	7.62	5.85E-06	2.65E-04
	Atopobium	Actinobacteria	1724.26	4.32	3.34E-05	1.14E-03
	Methanosphaera	Euryarchaeota	24.49	5.39	1.47E-04	3.99E-03
**Post- weaning (day 96)**						
Ileum digesta	Clostridium_sensu_stricto_1	Firmicutes	189.26	7.83	2.03E-05	4.01E-03

^1^The results presented for genera with FDR < 0.01; the complete results are presented in table S3c.

^2^L2FC: log2fold change: positive (+) value indicates a decrease in relative abundance in LA compared to ATB while negative value (−) indicates an increase in relative abundance in LA compared to ATB

LA: CTRL supplemented with *Lactobacillus acidophilus* BT1386 (LA; 2.5 × 10^8^ CFU/L milk replacer + 1 × 10^9^ CFU/kg starter feed)

ATB: chlortetracycline and neomycin (528 and 357 mg/L milk replacer, respectively), and chlortetracycline (55 mg/kg starter feed)

^3^FDR: P value corrected for False Discovery Rate.

Several genera were also found to be significantly DA between the two DFMs, and among them *Ruminobacter* (FDR = 1.72E-03) and *Lachnospiraceae*_UCG-008 (FDR = 3.71E-02) were the most significantly DA in pre- and post-weaning periods, respectively. *Ruminobacter, Moryella, Acetitomaculum* and *Prevotellaceae* UCG-001 were significantly reduced (FDR ≤ 7.96E-03) by SCB compared to LA (Table S3a).

### Predicted pathways of the relative changes due to treatments

To investigate the potential molecular pathways by which the microbiota adapted to treatments, we performed metagenomics contribution of the communities observed and differential analyses of predicted pathways between control and treatments for each site in pre- and post-weaning periods using the Kyoto Encyclopedia of Genes and Genomes (KEGG) database. A total of 6,205 KEGG orthologies (Table S4a) were predicted for all samples and assigned into 261 KEGG pathways (Table S4b). Metabolic pathway, biosynthesis of amino acids, ribosome, carbon metabolism and purine metabolism were the top 5 predicted pathways by relative abundance values for all GIT sites in both pre- and post-weaning periods (Table S4c). ECM-receptor interaction and AGE-RAGE signaling pathway in diabetic complications were only predicted for RuD, while Fc epsilon RI signaling pathway was uniquely predicted for IlD (Table S4c). Several pathways such as endocrine resistance, spliceosome, rap1 signaling, gap junction, and cytosolic DNA-sensing pathway were also uniquely predicted for CoM (Table S4c). The changes in abundance values for predicted pathways varied between treatments, site and day.

At the pre-weaning period, the SCB treatment significantly (p < 0.05) influenced 6 pathways (cell cycle, EGFR tyrosine kinase inhibitor resistance, bile secretion, Fanconi anemia pathway, mRNA surveillance pathway and oxytocin signaling pathway) in IlM and 5 pathways (caffeine metabolism, cAMP signaling pathway, steroid biosynthesis, proteasome and dopaminergic synapse) in RuD but had no impact on other GIT sites (Table 7) compared to CTL treatment. The LA treatment significantly (p < 0.05) impacted 4 pathways (caffeine metabolism, cAMP signaling pathway, steroid biosynthesis, proteasome and dopaminergic synapse) in RuD only, compared to CTL. The ATB treatment had diverse effects including significant (p < 0.05) changes to steroid hormone biosynthesis pathway in CoM, bile secretion and caffeine metabolism in IlM and cAMP signaling pathway, steroid biosynthesis and proteasome pathways in RuD compared to CTL (Table 7).

**Table 7:** Predicted KEGG pathways significantly changed by treatments at each gastrointestinal site in the pre- and post-weaning periods.

Gastrointestinal site	Treatment comparison^1^	Pathway name	log2FC^2^	P-value	FDR^3^
**Pre-weaning (day 33)**					
Colon mucosa	ATB	Steroid hormone biosynthesis	−0.484	3.95E-04	8.17E-02
Ileum mucosa	ATB	Bile secretion	−0.397	8.87E-05	1.84E-02
Rumen digesta	ATB	Caffeine metabolism	0.340	2.90E-06	6.00E-04
	ATB	cAMP signaling pathway	0.420	3.52E-05	3.64E-03
	ATB	Steroid biosynthesis	0.317	5.63E-04	3.89E-02
	ATB	Proteasome	0.310	1.76E-03	9.13E-02
	LA	Caffeine metabolism	0.336	7.79E-07	1.61E-04
	LA	cAMP signaling pathway	0.411	1.55E-05	1.61E-03
	LA	Steroid biosynthesis	0.304	4.16E-04	2.87E-02
	LA	Proteasome	0.299	1.34E-03	6.93E-02
	LA	Dopaminergic synapse	0.297	5.79E-03	2.00E-01
Ileum mucosa	SCB	Cell cycle	−0.358	5.25E-05	1.40E-03
	SCB	EGFR tyrosine kinase inhibitor resistance	−0.351	6.10E-05	1.40E-03
	SCB	Bile secretion	−0.400	7.90E-05	1.64E-03
	SCB	Fanconi anemia pathway	−0.353	1.66E-04	2.87E-03
	SCB	mRNA surveillance pathway	−0.271	7.44E-04	1.03E-02
	SCB	Oxytocin signaling pathway	−0.281	8.67E-04	1.12E-02
Rumen digesta	SCB	Caffeine metabolism	0.344	2.22E-06	4.60E-04
	SCB	cAMP signaling pathway	0.430	2.35E-05	2.43E-03
	SCB	Steroid biosynthesis	0.320	5.27E-04	3.64E-02
	SCB	Proteasome	0.315	1.48E-03	7.64E-02
	SCB	Dopaminergic synapse	0.320	5.24E-03	1.81E-01
**Post-weaning (day 96)**					
Colon mucosa	ATB	Caffeine metabolism	0.254	3.59E-04	7.43E-02
	ATB	Steroid biosynthesis	0.274	2.35E-03	1.83E-01
	ATB	cAMP signaling pathway	0.298	2.65E-03	1.83E-01
Ileum mucosa	ATB	RIG-I-like receptor signaling pathway	0.537	1.86E-05	3.85E-03
	ATB	D-Arginine and D-ornithine metabolism	0.449	1.90E-03	1.34E-01
	ATB	Butanoate metabolism	0.399	1.95E-03	1.34E-01
Ileum digesta	ATB	Thyroid hormone signaling pathway	−0.352	1.71E-05	3.54E-03
	ATB	Ether lipid metabolism	−0.451	5.63E-04	5.83E-02
Colon mucosa	LA	Caffeine metabolism	0.246	7.13E-04	1.48E-01
Ileum mucosa	LA	Cell cycle	−0.327	2.64E-04	6.95E-03
	LA	EGFR tyrosine kinase inhibitor resistance	−0.321	3.02E-04	6.95E-03
	LA	Oxytocin signaling pathway	−0.301	4.12E-04	8.53E-03
	LA	mRNA surveillance pathway	−0.271	8.03E-04	1.38E-02
	LA	Fanconi anemia pathway	−0.298	1.64E-03	2.43E-02
Colon mucosa	SCB	Caffeine metabolism	0.313	1.72E-05	3.55E-03
	SCB	Dopaminergic synapse	0.337	3.28E-03	1.16E-01
	SCB	cAMP signaling pathway	0.298	3.42E-03	1.16E-01
	SCB	Serotonergic synapse	0.301	3.87E-03	1.16E-01
	SCB	Steroid biosynthesis	0.266	3.94E-03	1.16E-01
Ileum mucosa	SCB	RIG-I-like receptor signaling pathway	0.505	5.57E-05	1.15E-02
	SCB	Steroid biosynthesis	−0.319	5.24E-04	4.72E-02
	SCB	Sphingolipid signaling pathway	−0.283	6.83E-04	4.72E-02
	SCB	D-Arginine and D-ornithine metabolism	0.461	1.42E-03	7.35E-02
	SCB	Metabolism of xenobiotics by cytochrome P450	−0.553	2.25E-03	7.81E-02
	SCB	Fructose and mannose metabolism	0.557	2.46E-03	7.81E-02
	SCB	Drug metabolism	−0.534	2.64E-03	7.81E-02
Rumen digesta	SCB	Thyroid hormone signaling pathway	−0.402	7.57E-07	1.57E-04
	SCB	Ether lipid metabolism	−0.524	5.78E-05	5.98E-03
	SCB	Cell cycle	−0.260	2.29E-03	4.37E-02
	SCB	Oxytocin signaling pathway	−0.250	2.41E-03	4.37E-02
	SCB	EGFR tyrosine kinase inhibitor resistance	−0.255	2.53E-03	4.37E-02
	SCB	mRNA surveillance pathway	−0.223	4.62E-03	7.36E-02
	SCB	Ascorbate and aldarate metabolism	−0.601	6.26E-03	8.19E-02
	SCB	Fanconi anemia pathway	−0.250	6.33E-03	8.19E-02
	SCB	Riboflavin metabolism	0.501	1.45E-02	1.77E-01

^1^Treatment CTRL: Control fed milk replacer followed by starter feed, ATB: CTRL supplemented with antibiotics (ATB) chlortetracycline and neomycin (528 and 357 mg/L milk replacer, respectively), and chlortetracycline (55 mg/kg starter feed). LA: CTRL supplemented with *Lactobacillus acidophilus* BT1386 (LA; 2.5 × 10^8^ CFU/L milk replacer + 1 × 10^9^ CFU/kg starter feed) and SCB: CTRL supplemented with *Saccharomyces cerevisiae* boulardii CNCMI-1079 (SCB; 7.5 × 10^8^ colony forming units (CFU)/L milk replacer + 3 × 10^9^ CFU/kg starter feed).

^2^L2FC: Log2fold change. Negative value indicate that treatment decreased the expression of pathway compared to control while positive value indicate that treatment increased the expression of pathway compared to control.

^3^FDR: False discovery rate corrected p-values.

At the post-weaning period, 5, 7 and 9 pathways were significantly (p < 0.05) changed by SCB compared to control in CoM, IlM and RuD, respectively (Table 7). The most significantly changed pathways by SCB during this period were caffeine metabolism (p < 1.72E-05), RIG-I-like receptor signaling pathway (p < 5.57E-05) and thyroid hormone signaling pathway (p < 7.57E-07) in CoM, IlM and RuD, respectively. Meanwhile, LA impacted the mucosa (IlM and CoM) only as it changed the abundance levels of caffeine metabolism (p < 7.13E-04) in CoM and of cell cycle (p < 2.64E-04) in IlM, EGFR tyrosine kinase inhibitor resistance, oxytocin signaling pathway, mRNA surveillance pathway and Fanconi anemia pathway (p ≤ 1.64E-03) in IlM. The ATB treatment significantly changed (p ≤ 5.63E-04) the abundance of thyroid hormone signaling pathway and ether lipid metabolism in IlD, cAMP signaling pathway in CoM and RIG-I-like receptor signaling pathway, D-arginine and D-ornithine metabolism and Butanoate metabolism in IlM.

## Discussion

Overall, the phylum Firmicutes was the most abundant in all GIT sites except the RuD where Bacteroidetes was the most dominant. Our results are supported by earlier reports of high relative abundance of Firmicutes in the GIT of pre-weaned Holstein calves^3^ or of Brazilian Nelore steer^31^. It is well documented that the bacterial community diversity pattern and composition differ across GIT sites^31,32^. In the current study, each GIT site was host to different bacteria community structures. In fact, we observed that CoM harboured a greater bacterial community diversity compared to other GIT sites. The colon is considered a fermentation tank for microbial fermentation of indigestible dietary substrates and the digesta is retained in the colon (large intestine) for a longer time compared to the small intestine (ileum), the colon being the hub of a more complex bacterial community^33^. In the colon, dietary fiber that escaped digestion in the upper digestive tract are broken down into short chain fatty acids and, the increased availability of short chain fatty acids promotes the growth of some bacterial in the lower GIT sites. Therefore, the increased bacteria growth is expected to account for the richness of bacteria in the colon^34^. The IlD had the lowest diversity compared to all other GIT sites. Peristaltic movements ensure a relatively short passage time through the ileum (3–5 h) by pushing the microbiota migration towards the large intestine, hence limited time for microorganisms to replicate and increase in numbers^35^ in IlD compared with other GIT sites investigated. Mucosa-associated microorganisms live in close contact with host cells; hence they execute different functions within the GIT compared to digesta microorganisms. This might account for the differences in diversity and composition of the ileum mucosa and digesta as seen in the current study.

As expected, alpha diversity measures were higher for post-weaning compared to pre-weaning. Likewise bacterial community composition was different in the post-weaning period as compared to the pre-weaning period in this study. In the early period of growth, the bacterial populations undergo dynamic changes in diversity and abundance as calf age^20^. Also, the bacterial communities in the GIT sites are significantly influenced by weaning^36^. The increased consumption of large amounts of solid feed and dietary shift from milk replacer with age has been given as the reason for age dependent increase in bacterial diversity^37^. The fermentation processes in the rumen is activated by the introduction of solid feed but there is a dramatic shift when milk is completely removed (weaning), greatly altering the composition of the ruminal and intestinal microbiomes^8^. The ruminal bacterial community is established before intake of solid food, but solid food arrival in turn shapes this community^38^. Dias *et al*.^39^ indicated that diet and age concurrently drive changes in the structure and abundance of bacterial communities in the developing rumen in calves. The PCoA plots in this study clustered according to period (pre-weaning and post-weaning) which is in line with Wang *et al*.^23^ who also indicated that bacteria communities clustered based on different age groups.

Previously, we recovered viable SCB and LA (total lactobacilli) throughout the GIT (rumen, ileum and colon) and feces of calves at the pre- and post-weaning periods^30,40^. Although growth performance (weight gain, feed intake and efficiency) was not affected by treatments^30^, calves were generally healthy and the treatments (LA and SCB) improved innate immune response (oxidative burst and phagocytosis) and markers of the acute phase reaction (CRP and SAA2), especially during weaning^40^.

The current study indicated that DFMs had less impact on bacterial diversity but more impact on bacterial composition in the GIT sites in calves. The greater diversity of SCB or LA compared to ATB (Table 1) might be linked to the differences in the mechanisms of pathogen clearance by ATB in the GIT. ATB eliminates pathogen growth by direct killing including neighbouring commensals, and therefore completely changing the ecological niche^41^. The diversity of the GIT has been shown to decrease both by short-term and long-term usage of antibiotics^42,43^. Decreased diversity by the use of ATB resulted in dysbiosis of the GIT microbiota leading to undesired effects, such as antibiotic-associated diarrhea^44^. The effects of DFMs on bacterial composition of GIT microbiota was site specific. Interestingly, major changes associated with DFMs were mostly found in the ileum and rumen compared to the colon (Tables 2 and 3), while a higher impact was observed at the pre-weaning period compared to the post-weaning period. The DA communities were composed of bacteria genera with beneficial effects to the host. The genera were phylogenetically related, suggesting a high level of functional redundancy, which is often associated with stable microbial assemblages resistant to pathogens^45^. Changes in microbial community compositions have been attributed to diet^46^. Since, LA and SCB treatments had different impacts, we will discuss the specific potential mechanisms for each DFM separately. For specific mechanisms, we will also focus our discussion on results reported at the genus level.

Perhaps, the most interesting results for SCB treatment was the significant reduction in the presence of *Tyzzerella_4* genus compared to control in IlM (Table 2). This genus belongs to *Lachnospiraceae* family and *Clostridia* class. Bacterial species of *Clostridia* class have the ability to form spores and some genera including *Tyzzerella_4* are linked to human diseases^47^. For instance, *Tyzzerella* and *Tyzzerella*_4 were associated to increased cardiovascular disease risk^47^. SCB treatment reduced the presence of *Streptococcus* compared to control in CoM. The pathogenic *Streptococcus* genus is widely distributed on the mucosal surfaces of the animal GIT^48^. Therefore, it suggests that SCB was able to eliminate numerous pathogens in the colonic mucosa compared to CTL or the other treatments. The microbiota influences the immune system by obstructing invading pathogens and can also support the growth and production of immune cells^49,50^. SCB also reduced the abundance of *Peptoclostridium* (*Clostridium difficile*) in IlM a major pathogen linked with infectious diarrhea^51^ (Table 2). In general, *Ruminococcaceae* are common digestive tract microbes that break down complex carbohydrates. SCB consumption positively influenced the establishment of *Ruminococcaceae* genera in the ileum of calves in this study. Brousseau *et al*.^52^ also found *Ruminococcaceae* bacterial family in the colon of pigs fed SCB and suggested that SCB had the potential as feed additives to modulate bacterial populations associated with GIT health^52^. *Ruminococcaceae*, actively degrades plants; it has carbohydrate-active enzymes, sugar transport mechanisms, and metabolic pathways for the degradation of complex plant materials^41,53^. As a member of the *Ruminococcaceae* family, *Ruminococcus* is a mucin-degrader and this probably enhanced mucus production which could be the reason for improved inflammatory responses in calves^54^. In a previous study, we also observed an increase in the concentration of markers associated with inflammatory response (acute phase proteins: CRP and SAA2) in calves fed LA or SCB^39^. Additionally, SCB also significantly increased the abundance of *Olsenella (Lactobacillus* reclassified as *Olsenella)* in IlM, a lactic acid bacterium that ferments carbohydrates to lactic acid^55^. This genus is bile-resistant and has the ability to utilise mucin^56^. Since *Olsenella* is a re-classification of lactobacillus species, its higher abundance supports our recent data in which we observed that SCB promoted the growth of total lactobacilli in the GIT of calves^33^. Surprisingly, the relative abundance of lactobacillus in LA treatment was similar to control in IlM at pre-weaning but decreased significantly (p = 8.93E-03) in IlD at post-weaning as compared to control. One possible explanation for this observation is that LA was probably a substrate for some other beneficial bacteria which disallowed its increase in some GIT sites even after supplemental feeding of LA. It is known that the product of one microbe is usually the substrate for another^57^.The genus Roseburia was also significantly (p = 7.01E-04) increased by SCB in IlM pre-weaning as compared to control. This is a commensal related genus producing short-chain fatty acids, particularly butyrate, which provides energy for cells in the GIT^58^, affects motility, maintains immunity, and has anti-inflammatory properties^59,60^. *Roseburia* may affect various metabolic pathways and could also serve as biomarkers for beneficial flora in GIT health^60^. This genus metabolizes dietary components that stimulate their proliferation and metabolic activities^60^. In mice, it has been shown that an increase in the abundance of *Roseburia* is linked to reduction of glucose intolerance^61^.

Many mechanisms of action of SCB have been directed against pathogenic microorganisms which include regulation of intestinal microbial homeostasis, interference with pathogens ability to colonize and infect the mucosa, modulation of local and systemic immune responses, and induction of enzymatic activity favoring absorption and nutrition. Consistent with the DA analyses, the major pathways changed by SCB treatment were in the IlM at the pre-weaning period. During this period, SCB significantly changed cell cycle, EGFR tyrosine kinase inhibitor resistance, bile secretion, Fanconi anemia pathway, mRNA surveillance pathway and oxytocin signaling pathway in IlM (Table 7). Since cell cycle and EGFR pathways are important for the regulation of cell proliferation, differentiation, growth, survival and motility, the SCB treatment might alter the bacterial abundance by influencing the genes or enzymes controlling these processes. Bile secretion pathway was also increased by SCB. This is a vital secretion essential for digestion and absorption of fats and fat-soluble vitamins in the small intestine^62^. In addition, bile is also an important route for elimination of excess cholesterol and many waste products, bilirubin, drugs and toxic compounds^63^. Bile acids appear to be a major regulator of the gut microbiota; and significant reduction in *Ruminococcaceae*^64^ has been related to low bile acid levels in the intestine^65^. Bile acids have been shown to have direct and indirect (through FXR-induced antimicrobial peptides) antimicrobial effects on gut microbes^66^.

Moreover, SCB treatment also altered the abundance of caffeine metabolism, cAMP signaling pathway, steroid biosynthesis, proteasome and dopaminergic synapse in the RuD. Steroid biosynthesis and proteasome are crucial pathways for lipid and protein metabolism while cAMP signaling pathway is important for second messengers signaling and have wide ranges of impact on cellular processes; therefore, it is not surprising that these pathways were impacted by the SCB treatment. However, it is not clear how caffeine metabolism pathway is related to SCB treatment in RuD.

Overall, health benefits of DFMs interaction can be classified into three categories^67^ as they can act directly within the GIT (level 1), they can also interact directly with the gastrointestinal mucus layer and epithelium (level 2) or they can have effects outside the GIT (level 3). The third level might reflect the effects of SCB on the dopaminergic synapse pathway. SCB might have impact on dopamine, an important and prototypical slow neurotransmitter in the mammalian brain, where it controls a variety of functions including locomotor activity, motivation and reward, learning and memory, and endocrine regulation^68^. However, the exact mechanisms are not clear.

At the post-weaning period, SCB also had an effect on five different pathways (caffeine metabolism, dopaminergic synapse, cAMP signalling, serotonergic synapse and steroid biosynthesis) and among them serotonergic synapse was the only pathway not affected by SCB in the pre-weaning period. Notably, serotonin (5-Hydroxytryptamine, 5-HT) is a monoamine neurotransmitter that plays important roles in physiological functions such as learning and memory, emotion, sleep, pain, motor function and endocrine secretion, as well as in pathological states including abnormal mood and cognition (http://www.genome.jp/kegg-bin/show_pathway?map=hsa04726&show_description=show). Interestingly, beside the effects on steroid metabolism, SCB increased the thyroid hormone signaling pathway (p < 0.0001) in RUD during the post-weaning period. Thyroid hormones are important regulators of growth, development and metabolism^69^; therefore it could be an important pathway involved in the SCB mechanism of action.

Generally, the LA treatment had less impact on the bacterial diversity (Table 1) but similar impact with SCB treatment on bacterial composition. At the pre-weaning period, LA also had greater impact on bacterial diversity in llM compared to other GITs sites. Similar to SCB treatment, *Tyzzerella_4* was the most significant genus decreased (FDR = 6.02E-08) and *Fibrobacter* was the most significant genus increased (FDR = 3.09E-02) by LA treatment in IlM (Table 3). However, some genera were significantly (FDR ≤ 2.33E-03) changed only by LA treatment including *Phascolarctobacterium, Prevotella_9* and *Candidatus_Soleaferrea*. Little is known about the functions of *Phascolarctobacterium*, and *Candidatus_Soleaferrea* genera in calf’s GIT but in human, *Phascolarctobacterium faecium* demonstrated a high colonization rate in the GIT^70^. In CoM, LA treatment also reduced *Turicibacter which* has been shown to possess putative immunomodulatory^71^ and invasive properties and may cause subclinical infections in piglets^72^.

In the post-weaning period, *Ruminococcus_*2, most significantly reduced by LA, has been shown to potentially associate with hyperinsulinaemia, intestinal permeability and hepatic inflammation in rats^73^. However, there is no information about the detrimental effects of this genus in calves.

In the pre-weaning period, LA treatment had significant impact on KEGG pathways only in the RuD which is similar to the impact of SCB during this period. However, at the post-weaning period, LA did not have significant impact on these pathways in RuD, but significantly changed caffeine metabolism pathway in the CoM and five pathways (cell cylce, EGFR tyrosine kinase inhibitor resistance, oxytocin signaling, mRNA surveillance and Fanconi anemia pathway) in IlM. Since these pathways were also significantly changed by SCB, we might assume similar potential mechanisms for SCB and LA in IlM.

The effects of antibiotics growth promoter on the bacteria community in the GIT system have been well documented. Several studies have shown that treatment with ATB altered the bacteria diversity^74,75^ as well as the bacteria composition^75,76^ in the GIT. The genera *Lactobacilli* and *C. perfringens* decreased in the ileum in broiler chickens fed low dose avilamycin and salinomycin^77^. Meanwhile the abundance of lactobacilli particularly *L. gasseri*, was increased by tylosin in the ileum of pigs^78^. However, we observed less impact of ATB on the GIT bacteria community at the pre- and post-weaning periods in this study. ATB significantly changed the bacteria composition in IlD and RuD only, at the pre-weaning period. Unlike SCB or LA, ATB had greatest impact on genera composition in the IlD and RuD, since it significantly changed the abundance of 34 and 24 genera in these sites, respectively, at the pre-weaning period. *Desulfovibrio* and *Ruminiclostridium_9* were the most significantly decreased or increased genera, respectively, by ATB treatment in IlD. Little is known about the roles of *Ruminiclostridium_9* in the GIT sites. Interestingly, no pathway was significantly changed by ATB treatment in IlD and RuD at the pre-weaning period. Notably, ATB also reduced streptococcus in the CoM and also significantly changed the abundance of steroid hormone biosynthesis pathways in the CoM at the pre-weaning period. In fact, *streptococcus* was the top most DA general in all three treatments (LA, SCB and ATB) in the CoM. Some species of the *Streptococcus* genera are pathogenic such as *Streptococcus pyogenes* and *Streptococcus pneumoniae*. However, *Streptococcus* was reduced in the treated samples with the largest reduction by SCB, followed by LA and ATB.

At the post-weaning period, *Sutterella* was DA by ATB. The genus *Sutterella* are commensals in the GIT with mild pro-inflammatory capacity in the human GIT^79^.

At post-weaning, ATB impacted steroid biosynthesis pathway in the CoM but targeted three different pathways including RIG-I-like receptor signaling pathway, D-Arginine and D-ornithine metabolism and butanoate metabolism pathways in IlM. RIG-I-like receptor proteins including RIG-I, MDA5, and LGP2 are expressed in both immune and non-immune cells. Upon recognition of viral nucleic acids, RIG-I-like receptor proteins recruit specific intracellular adaptor proteins to initiate signaling pathways that lead to the synthesis of type I interferon and other inflammatory cytokines, which are important for eliminating viruses.

The results from direct comparison of DA genera between treatments confirmed that the GIT microbiota was more sensitive to treatments in the pre-weaning period compared to the post-weaning period since most genera were significantly DA in the pre-weaning period (Tables 5, 6 and S3). Moreover, fewer genera and sites were affected when comparing LA vs. ATB than the comparison between SCB vs. ATB. This suggests that there were more diverse impacts of SCB compared to other treatments. Notably, Tyzzerella_4 (potential pathogenic genera) was the most significant DA genera in the pre-weaning period in both comparisons (SCB vs. ATB and LA vs. ATB) (Tables 5 and 6) suggesting differences in mechanisms by which the antibiotics (ATB) and DFMs (SCB or LA) can modulate pathogenic bacterial populations. Nevertheless, more studies are required to examine the distinct mechanisms by which DFMs impact the GIT of calves to enable development of effective DFMs.

The functional prediction analysis revealed more effects in the RuD contrary to data on diversity and abundance, which mostly influenced the ileum and colon. However, it is known that the level of abundance might not reflect the function of the bacteria and that roles played by the bacteria might be more important than abundance^80^, thus our data should be interpreted with caution.

In summary, the current data showed that site and day had an effect on bacteria diversity. However, the effect of treatment on bacteria diversity was not significant for most sites even though an increase in diversity was observed in the colon. The bacterial composition of the GIT microbiota was altered due to supplementation with the two DFMs with most DA genera found in the ileum. Both DFM treatments reduced some pathogenic bacteria genera such as *Streptococcus or Tyzzerella_4* and increased the potential beneficial bacteria, *Fibrobacter*. Other potential beneficial bacteria including *Rumminococcaceae UCG 005, Roseburia* and *Olsenella* were increased by SCB treatment only. The functional prediction via pathways enrichment analyses indicated that besides affecting the local pathways such as cell cycle, bile secretion, proteasome or cAMP signaling pathway both DFMs also impacted other pathways such as thyroid hormone synthesis or dopaminergic synapse in the brain pathway. Moreover, these DFMs also shared some common mechanisms with ATB; however they had more diverse target sites compared to the ATB which mainly targeted the colon microbiome. Although, this study indicates that DFM have site specific and age dependent effects on the calf gut microbiome, further system-omics related studies (meta-genomics, meta-transcriptomics, proteomics and metabolomics) are needed to better define the mechanisms related to these effects. Therefore, regional effects and age need to be taken into consideration when investigating the biological mechanisms by which DFMs affect the growth and development of calves at the early period of growth. Furthermore, the pre- and post-weaning samples were collected from different calves implying that some individual variation was expected to influence our results, thus our data should be cautiously interpreted.

## Materials and Methods

### Animal treatments and samplings

Animal management and use procedures were according to the Canadian Council on Animal Care^81^ and were approved by the animal care and ethics committee of Agriculture and Agri-Food Canada. Animal management procedures have been described in details previously^30^. Briefly, thirty two calves (2–7 days old) were randomly allocated to four treatments as follows: (1) Control (CTRL)- calves bucket fed with milk replacer (Goliath XLR 27–16, La Coop, Montreal, QC, Canada) at 6 L/day (2 L three times a day) for the first 4 days, and at 9 L/day (4.5 L twice a day) from day 5 to the end of weaning (day 53))and starter feed (Shur-Gain—Meunerie Sawyerville Inc., Cookshire-Eaton QC, Canada) fed *ad libitum* from day 8 of the experiment; (2) CTRL supplemented with *Saccharomyces cerevisiae boulardii* CNCMI-1079 (SCB; 7.5 × 10^8^ colony forming units (CFU)/L milk replacer + 3 × 10^9^ CFU/kg starter feed) (Levucell SB 20, Lallemand Animal Nutrition, Montreal, QC, Canada); (3) CTRL supplemented with *Lactobacillus acidophilus* BT1386 (LA; 2.5 × 10^8^ CFU/L milk replacer + 1 × 10^9^ CFU/kg starter feed) (Micro-Cell FS, Lallemand Animal Nutrition) and (4) CTRL supplemented with antibiotics (ATB) chlortetracycline and neomycin (528 and 357 mg/L milk replacer, respectively) pre-weaning, and chlortetracycline (55 mg/kg starter feed) (Vetoquinol Inc., Lavaltrie, QC, Canada) post-weaning. Calves were housed in individual pens, fed individually and had *ad libitum* access to water. The animal trial lasted for 14 weeks (experiment day 1 to 96). Weaning was initiated on day 43 by reducing the quantity of milk replacer offered by half every day and it was completed on day 53 when animals were able to eat 1 kg of starter feed per day. Four calves per treatment were euthanized on day 33 (pre-weaning) and another set of four calves per treatment on day 96 (post-weaning) to collect digesta samples from the rumen, ileum and colon, and mucosal samples from the ileum and colon. The pre- and post-weaning samples were collected from different calves. Digesta samples were aseptically collected placed in sterile tubes followed by storage at −20 °C until DNA isolation. Mucosal scrapings from intestinal tissues (colon and ileum) were collected using the inoculum method as described previously^82^ and stored at −80 °C until DNA isolation.

### DNA isolation and quantification

Samples were thawed and kept on ice during the extraction process. The digesta were disrupted using a high speed blender and mucosa samples as described above. DNA was isolated from the homogenate using the bead beating method with the ZR fecal DNA kit (Zymo Research Corp., Irvine, CA, USA) following manufacturer’s instructions. The quantity and purity of isolated DNA was measured using spectrophotometry (Nano Drop Technologies, Wilmington, DE, USA) and diluted to a final concentration of 30 ng/µl.

### Amplification of bacterial ribosomal DNA and sequencing

PCR primers targeting the 16S rRNA gene (V3–V4 region) were used to prepare amplicon libraries. Amplification of the 16S V3-V4 region was performed using sequence specific regions described previously^83^ in a dual indexed PCR approach. Briefly, the following generic oligonucleotide sequences were used for amplification: Bakt_341F-long AATGATACGGCGACCACCGAGATCTACAC[index1] TCGTCGGCAGCGTCAGATGTGTATAAGAGACAGCCTACGGGNGGCWGCAG and Bakt_805R-longCAAGCAGAAGACGGCATACGAGAT[index2] GTCTCGTGGGCTCGGAGATGTGTATAAGAGACAGGACTACHVGGGTATCTAATCC. The PCR was carried out in a total volume of 50 µL that contains 1X Q5 buffer (NEB), 0.25 µM of each primer, 200 µM of each dNTPs, 1 U of Q5 High-Fidelity DNA polymerase and 1 µL of template cDNA. The PCR started with an initial denaturation at 98 °C for 30 s followed by 10 cycles of denaturation at 98 °C for 10 s, annealing at 55 °C for 10 s and extension at 72 °C for 30 s, and 25 cycles of denaturation at 98 °C for 10 s, annealing at 65 °C for 10 s, extension at 72 °C for 30 s and a final extension step at 72 °C for 2 min. The PCR reactions were purified using the Axygen PCR cleanup kit (Axygen). Quality of the purified PCR product was checked on a DNA7500 BioAnalyzer chip (Agilent) and quantified using a Nanodrop 1000 Spectrophotometer (Thermo Fisher Scientific). Barcoded Amplicons were pooled in equimolar concentrations and sequenced on the Illumina MiSeq (paired–end 300 bases with two index reads). Library preparation and sequencing was performed by L’Institut de Biologie Intégrative et des Systèmes (IBIS), de Université Laval, Quebec City, Canada.

### Bioinformatics analysis

The downstream analysis of output fastq files was done using the pipeline of the open source software package QIIME^84^. Paired end reads were merged using FLASh^85^. Chimera detection was applied to the merged reads using Uchime^86^. The GOLD^87^ database was used for reference based detection. Taxomomic affiliation of the 16S data was studied using QIIME^84^. Demultiplexed and quality filtered sequences from pre-processing step were clustered into OTUs using VSEARCH^88^. An OTU (Operational Taxonomic Unit) was formed based on sequence identity with threshold defined at 0.97.After the clustering step, a representative sequence was picked for each OTU and a taxonomic identity was assigned to each representative sequence. The 16S database used was Greengenes while Uclust^86^ was used for taxonomic assignment. Multiple alignments of the representative OTU sequences were generated with PyNAST^89^, which aligns the sequences to 16S reference sequences. The relationship between sequences was studied by generating a phylogenetic tree with FastTree^90^ followed by computing UniFrac distances. A rarefaction curve for each sample was plotted (observed OTUs metric) in order to estimate the depth of sequencing for each sample and to choose the rarefaction threshold for all samples. Results were generated after the cumulative sum scaling (CSS)^91^ normalization method. The Amplicon-Seq pipeline provides taxonomic affiliation of data at different levels (Kingdom, Phylum, Class, Order, Family and Genus).

### Assessment of diversity and statistical analysis

Samples were rarefied for alpha-diversity calculations and rarefaction curves generated (Fig. S1) in order to eliminate the bias caused by the different sample sizes^92^. The OTU table was rarefied across samples to the lowest sample depth using QIIME based on the Messene Twister pseudorandom number generator. Alpha diversity estimators including Chao1, observed OTUs, Shannon, Simpson and Inverted Simpson (Invsimpson) were calculated for the overall bacterial community using Phyloseq^93^. Mean alpha diversity estimates for each site, day, treatment and treatment by site by day were compared using the two-sided t-test in R program^94^.

The dataset was also subsampled to the minimum^95^ to compare microbial composition between samples (β-diversity). Beta-diversity was measured by calculating the weighted and unweighted UniFrac distances^96^ using Phyloseq default scripts. Principal coordinate analysis (PCoA) was applied on the resulting distance matrices to generate two-dimensional plots. Permutational multivariate analysis of variance (PERMONOVA^97^) was used to calculate P-values and to test differences of β-diversity among treatment groups for significance.

### Bacterial Community Composition and differential relative abundance analyses

To investigate the relative abundance of the different genera, The MicrobiomeAnalyst^98^ was used to obtain the most prevalent bacteria genera within each site.

To investigate the effect of treatment on the different genera, we did a pair wise comparison between each treatment and control, GIT site and day (33 and 96). Different abundance at genus level was compared between treatments and control as well as among treatments using the Wald Test method of DESeq2^99^. The samples with OTU total count <10,000 were removed. The normalization step was done for each pair of comparison separately^100^ and taxa were considered significantly differentially abundant if p-corrected for false discovery rate (FDR) was <0.05. The FDR procedure is performed to reduce the type I error. In brief, this procedure includes the following steps: (1) uncorrected p-values are sorted in ascending order, (2) ranks to the p-values are assigned, (3) individual Benjamini-Hochberg critical p-values were calculated using the formula (i/m)q (i = the individual’s p-value rank, m = total number of tests, q = the false discovery rate). In this analysis, a q-value (FDR) of ≤0.05 was considered significant.

### Functional prediction and differential analysis of predicted pathways

The phylogenetic investigation of communities by reconstruction of unobserved states (PICRUSt)^101^ software was used for the prediction of functional genes of the classified members of the GIT microbiota resulting from reference-based OTU picking against Greengenes database. Predicted genes were then hierarchically clustered and categorized under Kyoto Encyclopedia of Genes and Genomes^102^ orthologs (KOs). Predicted KOs was then converted into their associated pathways. The differential analyses of predicted pathways were done in DeSeq. 2 and only pathways predicted for at least 5 samples were used as input data. The pathways were considered significantly differentially predicted if p was <0.05. Since the enrichment relied on human data, we used a relaxed threshold (uncorrected p-values) to get a better overview of the impact of treatments on pathways.

## Electronic supplementary material

Supplementary figure and table titles

Table S1

Table S2

Table S3

Table S4
