# Supplementary material for: Direct-fed microbial supplementation influences the bacteria community composition of the gastrointestinal tract of pre- and post-weaned calves
Source: Sci Rep. 2018 Sep 20;8:14147. doi: 10.1038/s41598-018-32375-5 (PMC6148029; doi:10.1038/s41598-018-32375-5)
Supplement: Supplementary file 1 — Supplementary figure and table titles [file 41598_2018_32375_MOESM1_ESM.pdf]

Supplementary Information

Direct-fed microbial supplementation influences the bacterial community composition of the gastrointestinal tract of pre- and post-weaned calves

Bridget E. Fomenky[1,2], Duy N. Do[1,3], Guylaine Talbot[1], Johanne Chiquette[1], Nathalie Bissonnette[1], Yvan P. Chouinard[2], Martin Lessard[1], and Eveline M. Ibeagha-Awemu[1]*

[1]Agriculture and Agri-Food Canada, Sherbrooke Research and Development Centre, Sherbrooke, Québec, Canada, J1M 0C8

[2]Département des Sciences Animales, Université Laval, Québec, Québec, Canada, G1V 0A6

[3]Department of Animal Science, McGill University, Ste-Anne-de Bellevue, Quebec, Canada, H9X 3V9

*Corresponding author:

Dr. Eveline Ibeagha-Awemu

Eveline.ibeagha-awemu@agr.gc.ca

Titles for supplementary tables

Table S1: The operational taxonomic units (OTU) present in the samples; from phylum to genus levels. (a) Bacteria phyla detected in all the samples (n = 159). (b) Bacteria families detected in all the samples (n = 159). (c) Bacteria classes detected in all the samples (n = 159). (d) Bacteria orders detected in all the samples (n = 159). (e) Bacteria genera detected in all the samples (n = 159)

Table S2: Genus relative abundance in the gastro-intestinal sites at pre- and post-weaning.

Table S3: The differential abundant comparison between treatments. (a) Significant differential abundant genera between SCB and LA treatments on day 33 (pre- weaning) and day 96 (post-weaning). (b) Significant differential abundant genera between ATB and SCB treatments on day 33 (pre -weaning) and day 96 (post-weaning). (c) Significant differential abundant genera between LA and ATB treatments on day 33 (pre- weaning) and day 96 (post- weaning)

Table S4: KEGG orthology and pathways predicted for all samples. (a) KEGG orthology; (b) Pathways abundance, and (c) Pathways relative abundance day site

Supplementary figure

Figure S1

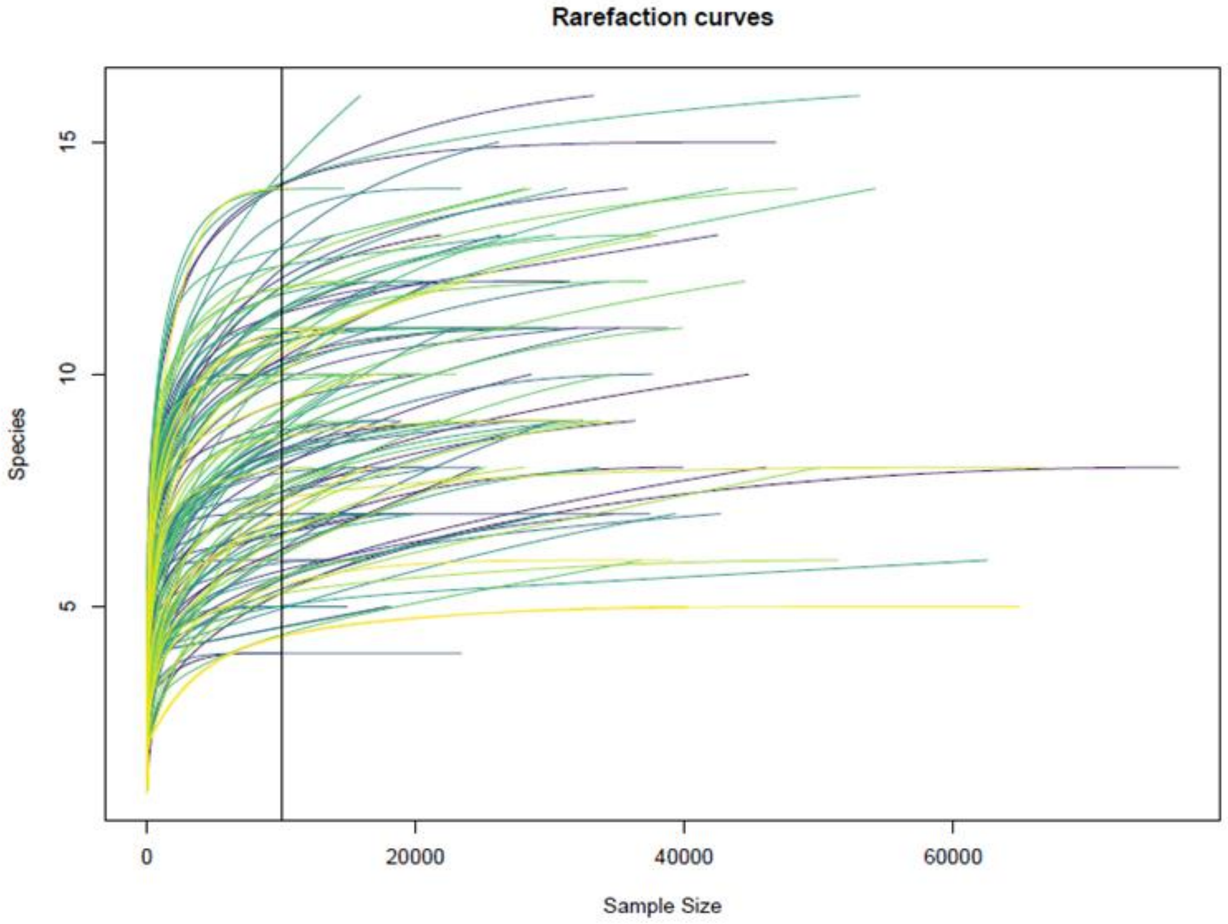

Figure S1: Rarefaction curves. Rarefaction curves (rarefied to the shallowest level for all sequences and plots of estimation of observed OTU against sequencing efforts). The y-axis shows the number of OTUs detected, and the x-axis the number of taxa in the sequence subset analyzed.
